# Mouse visual cortex contains a region of enhanced spatial resolution

**DOI:** 10.1038/s41467-021-24311-5

**Published:** 2021-06-29

**Authors:** Enny H. van Beest, Sreedeep Mukherjee, Lisa Kirchberger, Ulf H. Schnabel, Chris van der Togt, Rob R. M. Teeuwen, Areg Barsegyan, Arne F. Meyer, Jasper Poort, Pieter R. Roelfsema, Matthew W. Self

**Affiliations:** 1 Department of Vision & Cognition, Netherlands Institute for Neuroscience, Amsterdam, The Netherlands; 2 Donders Institute for Brain, Cognition and Behaviour, Radboud University, Nijmegen, The Netherlands; 3 Sainsbury Wellcome Centre for Neural Circuits and Behaviour, University College London, London, UK; 4 Department of Physiology, Development and Neuroscience, University of Cambridge, Cambridge, UK; 5 Department of Psychology, University of Cambridge, Cambridge, UK; 6 Department of Integrative Neurophysiology, Center for Neurogenomics and Cognitive Research, VU University, Amsterdam, The Netherlands; 7 Department of Psychiatry, Academic Medical Center, Amsterdam, The Netherlands

**Keywords:** Perception, Neural circuits, Sensory processing, Extrastriate cortex, Striate cortex

## Abstract

The representation of space in mouse visual cortex was thought to be relatively uniform. Here we reveal, using population receptive-field (pRF) mapping techniques, that mouse visual cortex contains a region in which pRFs are considerably smaller. This region, the “focea,” represents a location in space in front of, and slightly above, the mouse. Using two-photon imaging we show that the smaller pRFs are due to lower scatter of receptive-fields at the focea and an over-representation of binocular regions of space. We show that receptive-fields of single-neurons in areas LM and AL are smaller at the focea and that mice have improved visual resolution in this region of space. Furthermore, freely moving mice make compensatory eye-movements to hold this region in front of them. Our results indicate that mice have spatial biases in their visual processing, a finding that has important implications for the use of the mouse model of vision.

## Abstract (web summary)

The representation of space in mouse visual cortex was considered to be relatively uniform. The authors show that mice have improved visual resolution in a cortical region representing a location in space directly in front and slightly above them, showing that the representation of space in mouse visual cortex is non-uniform.

## Introduction

The organization of the mouse cortical visual system resembles that in primates because it is organized into a primary visual area (V1), surrounded by a number of retinotopically organized higher visual areas^1–5^. In primates, however, the early visual areas have a foveal confluence^6^, a greatly expanded region representing the central 2° of visual space. Mouse retinas lack a fovea, the region of the retina with greatly enhanced photoreceptor density in comparison to the periphery, and consequently mouse visual cortex does not possess a foveal representation. This has implications for the use of the mouse as a model for the human visual system. For example, it might be unnecessary for mice to move their eyes to bring interesting objects into a specialized region of the retina for more detailed analysis. However, previous studies suggested that the mouse retina is not entirely uniform^7,8^ and that the representation of space in mouse visual cortex may be enlarged for particular regions of the visual scene^3,4,9^. To determine whether there might be regions with enhanced spatial resolution in mouse visual cortex, we investigated the representation of space using population receptive field (pRF) mapping^10,11^. pRF mapping is a forward-modeling technique in which the Gaussian profile that best fits the response of a point in cortex to mapping stimuli is taken as an estimate of the aggregate receptive field (RF) at that point (Supplementary Fig. 1). The pRF approach has the advantage over traditional phase-encoded retinotopy that it allows estimates to be made of both the location and size of the aggregate RF. The maps of pRF size revealed a region, which crossed visual area boundaries, containing considerably smaller pRFs than surrounding regions. We pursued the neural organization of this region using electrophysiology and two-photon calcium imaging and found that the smaller pRFs were due to a decrease in the spatial scatter of single-cell RFs in a region in front of and slightly above the mouse, combined with increased cortical magnification in binocular regions of space. Mice had improved spatial resolution in this region of space and freely moving mice made eye movements to compensate for changes in head position to hold the region of smaller pRF size in front of them, slightly above the horizontal plane.

## Results

### Wide-field imaging reveals a region with small pRFs

**Fig. 1:**
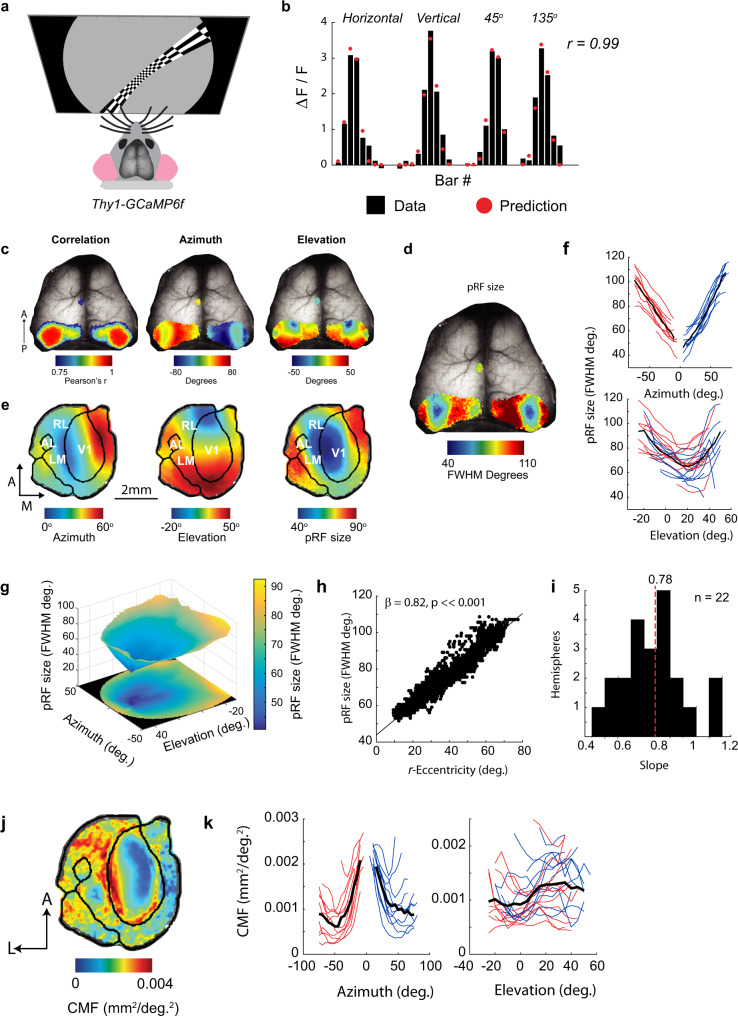
Wide-field calcium imaging reveals a cortical region with small pRF size in mice. **a** Calcium signals were imaged through the cleared skull of Thy1-GCaMP6f mice viewing checkerboard bars of different orientations and positions. **b** The change in fluorescence in response to 31 different bar stimuli from an example pixel (black bars). The predictions of the pRF model are shown as red dots. Pearson’s correlation between the model and the data of the example pixel was 0.99 (*p* ≤ 0.001, H0: *r* = 0, two-tailed). **c** Example cortical maps showing the correlation of the pRF model (left panel), the azimuth of the best-fitting Gaussians (middle panel), and the elevation of the Gaussians (right panel) overlain on the brain imaged through the skull. The maps are thresholded at a correlation coefficient of 0.75. **d** Maps of pRF size (the full-width at half-maximum of the best-fitting Gaussian). A region of smaller pRF size was observed in left and right visual cortex in all imaged mice. **e** Average azimuth, elevation, and pRF size maps from 17 mice (only the left hemisphere was imaged in these mice to examine visual area boundaries at higher resolution). The boundaries of V1 and other visual areas were identified using field-sign analysis and are overlaid on the maps as black lines. The maps of individual mice were recentered on V1 and resized by the size of V1 before averaging. The region of small pRF size is centered on the lateral border of V1 and extends into higher lateral visual areas LM and RL. **f** The relationship between pRF position and size. Azimuth and elevation values from individual pixels were binned. The red/blue lines show data from the right/left hemispheres of 11 mice who were imaged bilaterally. The black line shows the average across mice. **g** The size of pRFs can be visualized as a 3D surface and is approximately linearly related to the distance from a point in space at 0° azimuth and 20°elevation that we refer to as the focea. The surface was fit to the azimuth and elevation values using linear interpolation. **h** An example from a single hemisphere showing that pRF size is linearly related to the spherical angle between the pRF center and the focea, which we refer to as r-eccentricity. Linear regression H0: *β* = 0 (two-tailed). **i** The distribution of the slope-coefficients, *β*, across all 22 hemispheres of bilaterally imaged mice, the average slope is shown by the dashed red line. **j** The average map of cortical magnification factor (CMF) across 17 unilaterally imaged mice. **k** The relationship between CMF and pRF location in bilaterally imaged mice (22 hemispheres imaged mice). CMF was highest at azimuths close to the vertical meridian (left panel), and increased in regions of azimuth below 30° (i.e., binocular regions). The relationship between CMF and elevation was less clear and variable across animals (right panel).

We measured the calcium responses to pRF mapping stimuli (high-contrast checkerboard bars) through the intact skull of awake mice expressing the genetically encoded calcium indicator GCaMP6f^12^ (Fig. 1a). The responses from each pixel were fit with a pRF model in which the pRF is assumed to be a two-dimensional Gaussian function (Supplementary Fig. 1). The pRF model provided an excellent fit to the average calcium signals obtained in response to checkerboard mapping stimuli (Fig. 1b) and the resulting maps of pRF azimuth and elevation closely resembled those of previous studies using traditional phase-encoded retinotopy (Fig. 1c). The maps of pRF size (Fig. 1d) revealed pRFs with sizes of approximately 40° to over 100° of visual angle (the full-width at half-maximum of the Gaussian function, FWHM). These estimates are considerably larger than single-cell RFs measured using electrophysiology that typically lie in the range of 10°–20°^13^. Surprisingly, there was a clearly organized gradient of pRF sizes. A distinct region with smaller pRF sizes was surrounded by regions preferring larger sizes (Fig. 1d). The region with small pRF sizes was centered on the lateral border of the primary visual cortex (V1), but extended into neighboring areas including LM, AL, and RL (unilaterally imaged mice, see Methods, Fig. 1e, and Supplementary Fig. 2) in a region of cortex representing space in the binocular zone in front of the mouse. We investigated the relationship between the pRF location and size by binning the azimuth and elevation values of all V1 pixels and averaging pRF sizes per bin. pRFs were smallest at azimuths of 0° (i.e., directly in front of the mouse) and at an elevation of 20° above the horizontal plane (Fig. 1f). When visualized as a 3D surface (Fig. 1g), a region with small pRFs at approximately [0°, 20°] (Azimuth, Elevation) was evident, with pRF sizes increasing at larger distances from that point. We refer to the [0°, 20°] point hereafter as the focea to distinguish it from the fovea in primates. The term “fovea” refers to a region of enhanced photoreceptor density in the primate retina, which is absent in mice. We therefore propose to use the term “focea” to denote a cortical specialization for a particular region of the visual scene. The surface was well-fit (*r*^2^ > 0.9 for left and right hemispheres of V1) by a linear model (see Methods) in which the pRF size was proportional to the distance to the focea (hereafter denoted as recentered eccentricity or r-eccentricity; r-eccentricity is the angle between the center of the pRF and the 0° azimuth, 20° elevation point, in a spherical coordinate system centered on the mouse, see Methods), with an average slope of 0.78 (pRF size per r*-*eccentricity, both in °; Fig. 1h, i). Minimum pRF size fits were obtained at [azimuth = −1°, elevation = 20°] in the left hemisphere and [azimuth = −1°, elevation = 17°] in the right hemisphere. The relationships between azimuth, elevation, and pRF size were qualitatively similar in the higher visual areas that we could reliably map using this technique (Supplementary Fig. 3). pRF sizes were larger in LM, RL, and PM than in V1, but the smallest pRFs were still centered on approximately [0°, 20°]. In accordance with previous results^3,4,9^, the cortical magnification factor (CMF) was higher close to the vertical meridian (azimuth = 0°) but we did not observe an increase in the cortical magnification at an elevation of 20°, the locus of the smallest pRF size (Fig. 1j, k). While CMF was higher in binocular regions of cortex, binocularity by itself was a poor predictor of pRF size (Supplementary Fig. 3b). We used a spherical correction to correct for flat-screen distortion (see Methods) and excluded that the small pRFs at the focea were caused by this correction (Supplementary Fig. 4).

### The size of the spiking receptive fields of cells varies weakly with eccentricity

**Fig. 2:**
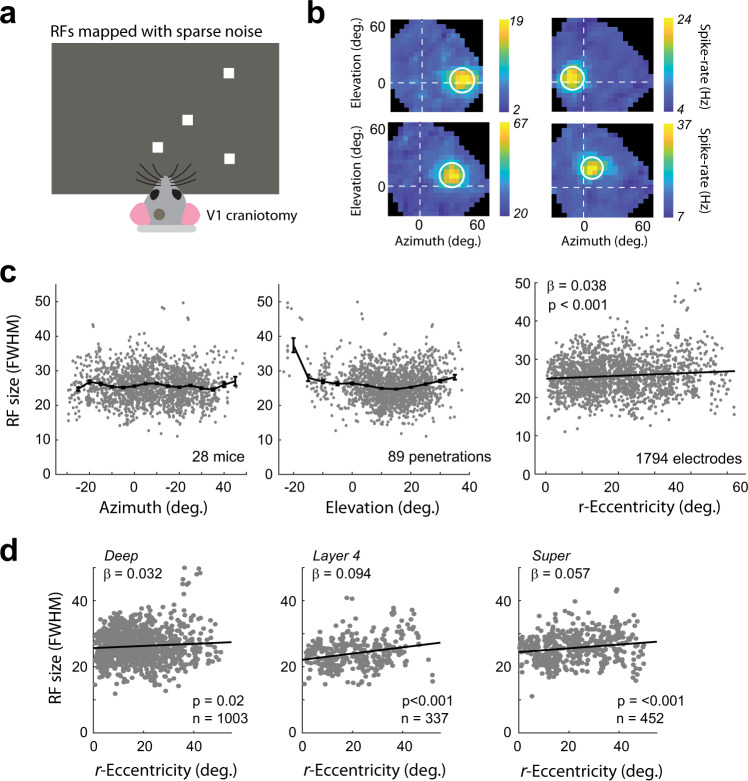
Electrophysiological analysis of RF size. **a** A total of 28 awake Thy1-5.17-GCaMP mice viewed sparse-noise RF mapping stimuli while multi-unit neural activity was measured across the different layers of V1 using a linear probe. **b** Four example RFs showing the average change in spiking in response to each check position. The white circle denotes the FWHM of the best-fitting Gaussian. **c** The azimuth (left panel) and elevation (middle panel) of the RFs across all animals. The binned average (bin size = 5°) is shown as the black line. RF size was relatively constant across different azimuths/elevations with the exception of one penetration at a more negative elevation where RF sizes were larger. Right panel: the relationship between r-eccentricity (angle between the RF center and the focea in a spherical coordinate system) and RF size showed a weak, but significant positive relationship (Linear regression. H0: *β* = 0 (two-tailed)). Error bars indicate SEM. **d** The slope of the relationship between r-eccentricity and RF size was significant in the individual layers. The slope did not differ significantly across laminar compartments (ANCOVA, *F*_2,1786_ = 3, *p* = 0.05).

The signals measured using wide-field calcium imaging contain contributions from different cellular compartments (e.g., dendritic arbors, axonal signals) and layers of cortex. To examine the relationship between r-eccentricity and the RF size of spiking activity in the different cortical layers, we carried out laminar electrophysiological recordings in 28 awake mice in V1 (1794 recording sites in 89 penetrations) (Fig. 2a). RFs were measured using standard sparse-noise stimuli and were fit with a 2D Gaussian function (see Methods, Fig. 2b). Surprisingly, we observed only a very weak relationship between the RF size and the azimuth and elevation of the RF (Fig. 2c). The slope of the regression of RF size on r-eccentricity was considerably shallower than in the wide-field data, though due to the large size of the dataset it was still significantly greater than zero (linear regression: *β* = 0.038; *r*^2^ = 0.009, *p* < 0.001). We also observed that cells with RFs located at very negative elevations (lower visual field) had much larger RFs, but this region was rarely targeted during our recordings and may have impinged upon the blind-spot of the mouse. The regression slopes were similar in the different layers (ANCOVA; interaction between layer and eccentricity: *p* = 0.05, Fig. 2d). In spite of the slight increase of RF size with eccentricity in the electrophysiological data, the average RF size at the larger eccentricities remained below 30°, implying that it cannot account for the large increase of pRF size with eccentricity in the wide-field data (Fig. 1h, i).

**Fig. 3:**
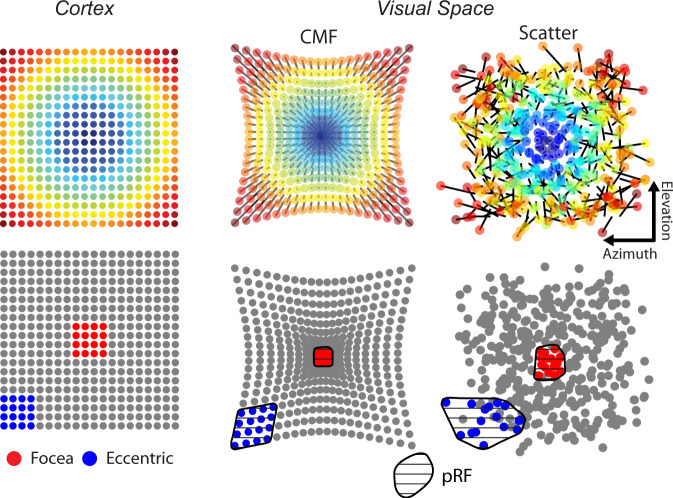
Conceptual models of pRF size. Upper row: two conceptual models of V1 that could account for the influence of r-eccentricity on pRF size in the wide-field data. The top left panel shows the position of model cell bodies in V1. The other panels show models in which RF positions are displaced purely by changes in cortical magnification factor (CMF), with lower magnification at higher eccentricities (middle) or by increased RF scatter at larger eccentricities (right). The black lines connected to each RF illustrate the displacement of the RF. Lower row: two equally sized analysis windows were drawn on the cortex, one at the foceal representation (red) and one in the periphery (blue). The RFs of cells within the analysis window are shown colored in the right panels. An estimate of the pRF can be made by taking the convex hull (shaded region) of the RF positions in space. Both the CMF and scattering model result in smaller pRFs at the focea (compare the areas of the red and blue regions).

How can these two datasets be reconciled? We generated conceptual models of mouse V1 (Fig. 3) to explore two non-mutually exclusive possibilities: (1) our wide-field data and previous studies^3,4,9^ suggest that the CMF is highest close to the vertical meridian. If a larger region of cortex is dedicated to processing the focea compared to other parts of the visual scene, then pRFs, as measured by wide-field imaging, would be smaller at the focea. (2) The representation of visual space is not perfectly organized and RFs show scatter at a local level^14,15^. If the representation of visual space is better organized at the focea, then neighboring RFs will exhibit less scatter and the aggregate pRF, as measured by wide-field imaging, will be smaller than at larger eccentricities. To independently determine the contribution of the two models to pRF size, it is necessary to first estimate the cortical magnification function at different locations in visual space and then examine the residual scatter of RFs from their location as predicted by the function.

### Two-photon imaging reveals lower scatter of RFs in the focea

**Fig. 4:**
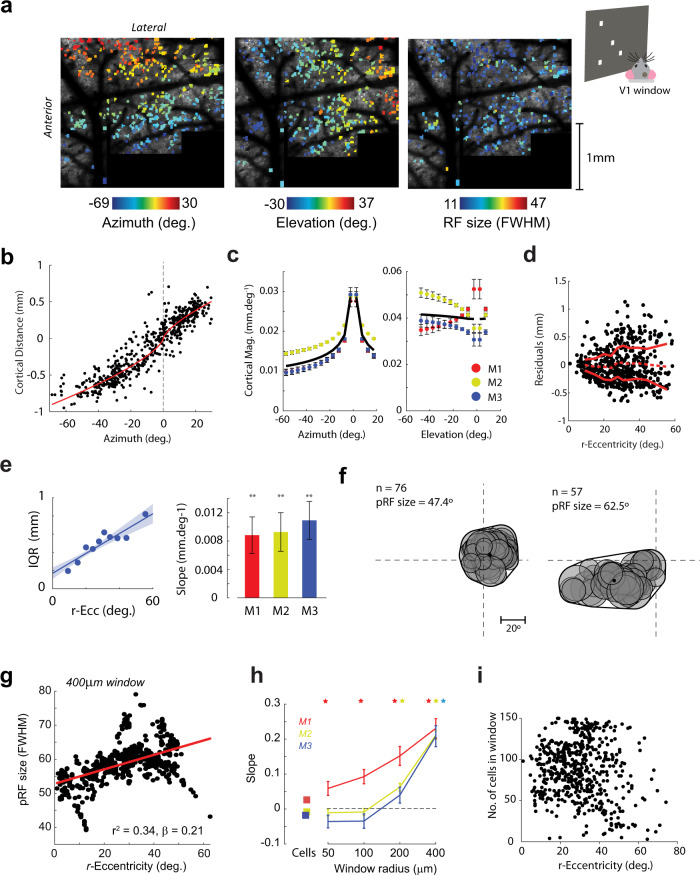
The region of small pRF size is due to higher cortical magnification and decreased RF scatter. **a** Tiled two-photon images from an example mouse covering almost the entirety of V1. The mouse viewed a screen placed at an angle of 30° so that the left visual field could be mapped with sparse noise. Cells for which we could reliably measure the RF (*r*^2^ > 0.33, BVI < 1, see Methods) are shown in color according to their preferred azimuth (left), elevation (middle), and RF size (right). The mean image of cortex is shown in the background. **b** An example relationship between the azimuth of the RF and the distance of the cell body from the foceal representation. The red line shows the fit of an exponential function. The cortical magnification factor (in mm/deg) can be estimated by the slope of this fit. **c** CMF estimates in the azimuth and elevation directions. The black line is the average across three mice. **d** RF scatter was estimated by examining the residuals of the RF positions from the exponential fit. The solid red lines indicate the interquartile range of the residuals and the dashed line the mean residual value in 10° sliding windows. r-eccentricity is the spherical angle between the RF center and the focea. **e** (Left panel) An example linear regression of the interquartile range of the residuals on r-eccentricity. The shaded region shows ± SEM (right panel). The slopes were significantly positive in all three mice (bootstrap test, one-tailed) indicating increased scatter of the residuals with distance from the focea in visual space. **p < 0.01. **f** Two example pRFs constructed from the single-cell data. For every cell falling within an analysis window (400 µm radius in this example), the Gaussian RF fit was projected into visual space (gray circles). The convex-hull of the resulting region and its area were used to estimate the pRF and its size. *n*, number of cells contributing to the pRF. **g** Example linear regression of pRF size on r-eccentricity in M2. **h** The slope values (*β*-coefficients) from three mice as a function of window radius. Asterisks, slopes significantly greater than zero (*t*-test, *p* < 0.05, two-tailed), error bars indicate 1 SEM. The slopes determined from individual cell RFs (without computation of aggregate RFs) are shown as square symbols. **i** There was no significant relationship between r-eccentricity and the number of cells in the analysis window (*p* > 0.05, linear regression, two-tailed).

Are the smaller pRFs at the focea in the wide-field imaging data due to increased CMF, decreased RF scattering, or a combination of both effects? To address this question we examined the positional scatter of V1 RFs with two-photon imaging in layer 2 of three Thy1-GCaMP6f mice implanted with a cranial window. We measured the RFs with sparse-noise stimuli as described above for the electrophysiological experiments. We tiled most of V1 with successive imaging fields and stitched the images together into a single large, high-resolution representation (Fig. 4a). We first restricted our analysis to RFs measured from individual cell bodies (see Methods). There was no obvious relationship between the azimuth, elevation, or r-eccentricity of the individual cells’ RF and the RF size (Supplementary Fig. 5), consistent with the weak relationship observed in the electrophysiological data. The relationship between the position of a cell’s RF in visual space and the position of its cell body in cortex can be approximated by an exponential function^16^, and the slope of this relationship gives an estimate of the cortical magnification function (Fig. 4b) (see Methods). We estimated the cortical magnification function separately for the azimuth and elevation directions (Fig. 4c). Magnification in the azimuth direction varied by a factor of two from approximately 0.01 mm/deg in the periphery to 0.02–0.03 mm/deg in the binocular zone (Fig. 4c), confirming the findings from the wide-field data (Fig. 1k). In the elevation direction, CMF was relatively constant with a value of ~0.04 mm/deg. To estimate the scatter of RF positions we examined the residuals between the exponential fits and the observed RF positions (Fig. 4d). The residuals showed a “fanning-out” profile with increased variability at larger eccentricities. We quantified RF scatter using the interquartile range of the residuals in sliding windows between 0° and 50° eccentricity. In all three mice, there was a significant linear relationship between RF scatter and eccentricity (Bootstrap test, see Methods, all mice: *p* < 0.01, Fig. 4e) suggesting that the representation of visual space is better organized at the focea than elsewhere. We examined whether RF scatter also differed between the monocular (azimuth >±15°) and binocular (azimuth <±15°) regions of cortex. However, the distribution of the residuals was very similar in both regions (bootstrap test, all three mice *p* > 0.05, see Methods). We next examined whether the decreased scatter at the focea could explain the relationship between r-eccentricity and pRF size observed in the wide-field data, using a method that we will refer to as “scatter analysis.” In this analysis we estimated pRFs by pooling single-cell RFs within analysis windows of different sizes drawn on the cortical surface. The pRF was estimated as the convex-hull of the single-cell RFs in visual space (Fig. 4f) (see also Methods). We found a clear and consistent linear relationship between r-eccentricity and pRF size (an example is shown in Fig. 4g). The slope of the relationship became steeper with larger analysis windows (*p* < 0.05 for all three mice at window sizes of 400 μm radius; Fig. 4h). We ensured that this relationship was not driven by differences in the number of cells within the windows by including cell number and the square-root of cell number in the regression analysis. Furthermore, there was no significant relationship between cell number and eccentricity (*p* > 0.05, linear regression) (Fig. 4i).

**Fig. 5:**
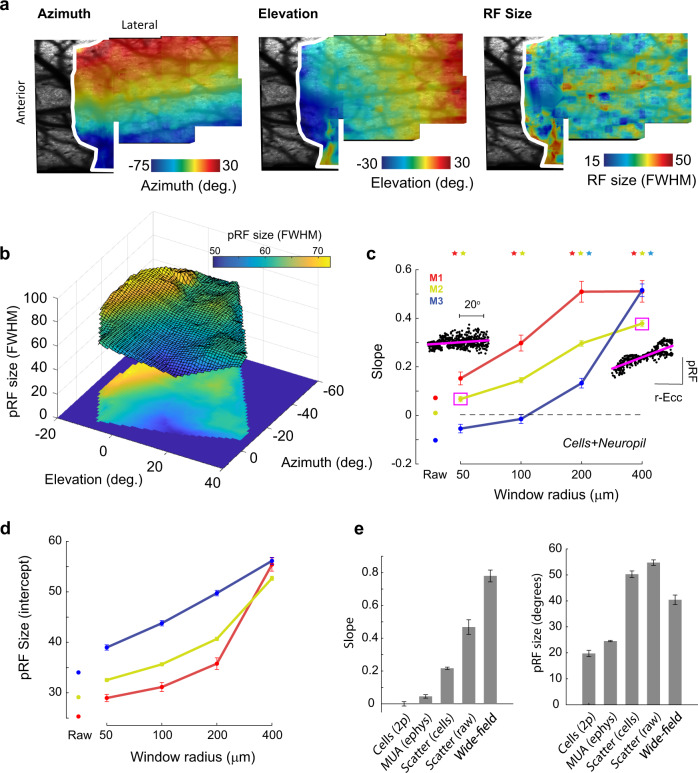
pRFs generated from raw two-photon images show stronger scattering. **a** Example maps of azimuth and elevation generated from the (smoothed) raw two-photon images show a clear retinotopic organization in agreement with the RFs measured for the individual cells. The white border indicates the boundary of V1 as determined by field-sign analysis (Methods). The map of RF size (right panel) showed no clear organization at this level of spatial detail. These images form the input into the scatter analysis. **b** Measures of pRF size obtained from performing the scatter analysis on the retinotopic maps shown in (**a**), a window size of 400 μm was used to generate this image. The smallest pRFs are in the region representing the focea. **c** The slope of the regression of pRF size on r-eccentricity for the raw image data approached the values from the wide-field data in Fig. 1i for the larger analysis windows. Example regression fits for mouse M2 are shown in the insets. Asterisks mark significant values, *p* < 0.05, *t*-test, two-tailed, and the error bars indicate 1 SEM. **d** The intercept term of the regression gives the expected pRF size at the focea. This approached the minimum values observed in the wide-field data (approximately 40–50°) only at window sizes of 200–400 μm radius, suggesting that windows of this size best capture the signals that are measured in the wide-field data. The pattern was consistent across mice (colored lines). Error bars indicate 1 SEM. **e** Summary of the slope (left) and intercept (values) for the different techniques. The results indicate that the small pRFs in the focea are caused by reduced the scatter of RFs across cells. Techniques that measured individual cells or small multi-units (electrophysiology, two-photon cell analysis) did not find strong relationships, whereas those that measured activity pooled over many cells (two-photon scatter analyses and wide-field analysis) found a relationship. The data from the cell+neuropil analysis (i.e., raw) had slope values closest to the wide-field data. The values for the scatter analyses are taken from the 400 μm radius analysis windows. Error bars indicate 1 SEM across animals.

The results of the scatter analysis using cell bodies suggest that decreased RF scatter contributes to the smaller pRFs observed in the foceal representation in the wide-field data. However, the slope of the regression reached a value of ~0.25 for a window radius of 400 μm (Fig. 4d), which is smaller than the slope of 0.78 in the wide-field data (Fig. 1i). We therefore considered further possible sources of scatter that were not captured by the analysis of cell bodies. As wide-field signals also contain contributions from neuropil, we reanalyzed the two-photon data, but this time without isolating individual cells (see Methods). We measured the RFs of individual pixels in the smoothed raw images, thereby including contributions from both cell bodies and neuropil. As expected, the RFs from individual pixels in the raw image data were organized into clear retinotopic maps, and the pixel RF size at this fine spatial scale was relatively constant across azimuths, elevations, and eccentricities (Fig. 5a and Supplementary Fig. 5). We next generated pRFs by pooling calcium activity within windows of different sizes and examined the relationship between the r-eccentricity of the pRF and its size (scatter analysis; Methods). There was a clear linear relationship between r-eccentricity and pRF size with pRF sizes being smallest close to the focea (Fig. 5b). The slope of the relationship was larger than that for the cell body data and approached the slope values for the wide-field data at 400 μm window sizes (Fig. 5c). Furthermore, at this window size, the size of the pRFs was similar to that in the wide-field data (Fig. 5d). We examined the level of scatter in the neuropil RF maps and found that, while the neuropil maps were generally less scattered than the single-cell maps, the relationship between scatter and r-eccentricity was steeper than for single cells (Supplementary Fig. 6). The results indicate that RF scatter of cells and neuropil provide major contributions to the relationship between r-eccentricity and pRF size as observed in the wide-field data. The scatter in the RF position of cell bodies^14^ accounts for a fraction of the increase in wide-field pRF with r-eccentricity and the scatter of the RF positions of neuropil accounts for a further fraction (Fig. 5e).

### Functional significance of the focea

**Fig. 6:**
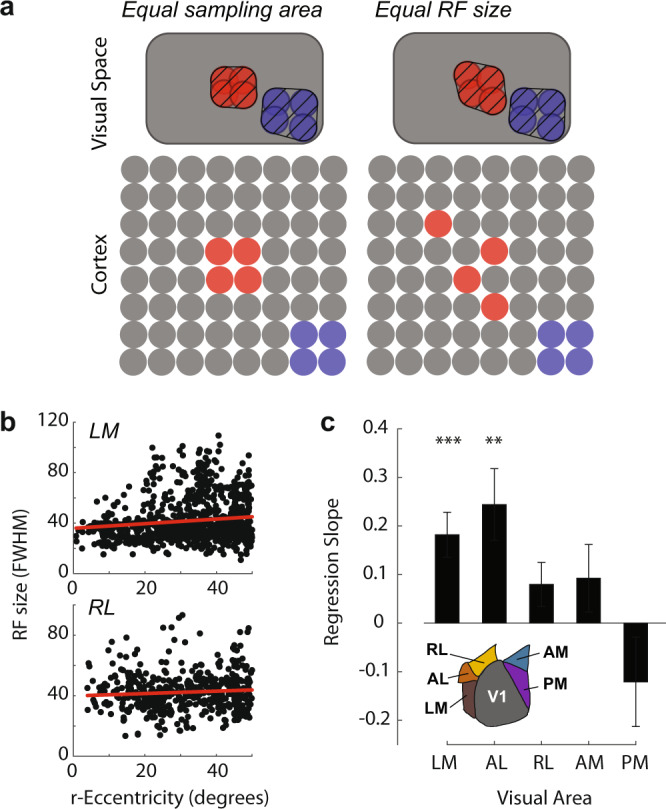
Receptive fields in three higher visual areas are larger at greater eccentricities. **a** If neurons at all retinotopic positions in higher visual areas sample homogeneously from equal-sized regions of V1 (left panel), then neurons sampling from the foceal representation (red cells) will have smaller receptive fields (red cross-hatched region) than cells sampling from more scattered representations in the periphery (blue cells). Alternatively, neurons at different retinotopic positions in higher areas may use different V1 sampling strategies (right panel), which could counteract the reduced scatter at the focea to equalize RF size across eccentricities (compare the red vs blue cross-hatched region). **b** The relationship between r-eccentricity and single-cell RF size in LM (*n* = 959 cells) and RL (*n* = 505 cells) measured using two-photon imaging. **c** RF size increased with eccentricity in LM and AL. Asterisks indicate regression slopes that were significantly greater than zero: **p < 0.01, ***p < 0.001 (Linear regression, H0: *β* = 0, two-tailed).

If the decreased scatter of RFs in the focea is of functional significance, one might predict that it causes smaller RFs in downstream visual areas. Cells in higher visual areas pool their inputs from a circumscribed region of V1. Less RF scatter at the focea could lead to smaller RFs in single cells in higher visual areas (Fig. 6a), which, in turn, might result in a higher visual acuity. We therefore examined RF sizes of single cells in higher visual areas with two-photon imaging. We imaged five higher visual areas (LM, AL, RL, AM, and PM) and mapped single-cell RFs as described above. As expected, RFs were generally larger in the higher areas than in V1. The size of the RFs in areas LM (Fig. 6b) and AL increased with r-eccentricity with slope values similar to that obtained using the scatter analysis on cell bodies in V1 (Fig. 4g, h) (LM: *β* = 0.18, *p* < 0.001. AL: *β* = 0.24, *p* < 0.01). RF sizes in areas RL, AM, and PM, however, did not exhibit a significant relationship with eccentricity (all *p* > 0.05) (Fig. 6c). Our results indicate that RF sizes of neurons in areas LM and AL increase with r-eccentricity just as the pRFs do in V1 but that RF sizes in RL, AM, and PM are constant across eccentricities.

**Fig. 7:**
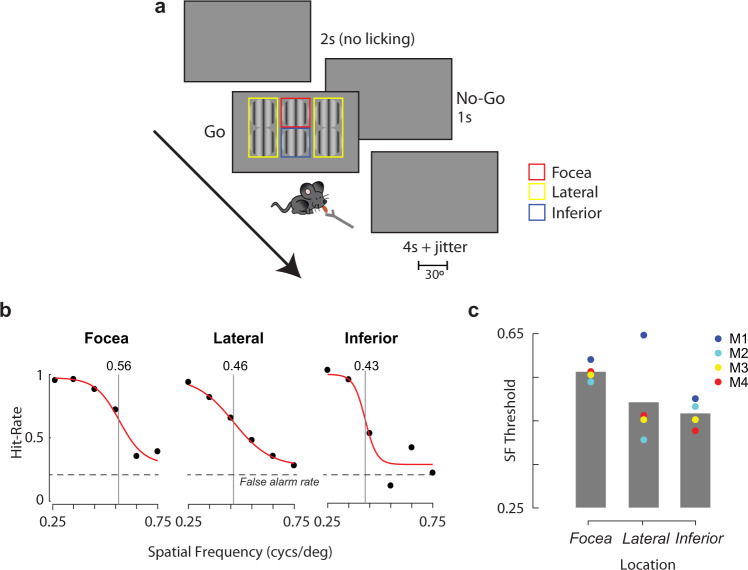
Visual acuity is higher at the focea. **a** Contrast detection task. Mice were trained to lick upon detection of a grating stimulus presented at one of 6 different spatial locations (“go” trials). The locations were grouped into three conditions indicated by the colored squares (not visible to the mouse): focea (red), lateral (yellow), and inferior (blue). On no-go trials no stimulus was presented and mice were trained to refrain from licking. False alarms were punished with timeouts. **b** Fraction of correct “go” trials for different spatial frequencies of the grating for three different spatial locations for an example mouse. “Focea” indicates stimuli presented in the upper-central visual field at (0° azimuth, 20° elevation), “Lateral” shows responses pooled over the four stimuli at lateral locations (±35° azimuth, −10°/+20° elevation), and “Inferior” for stimuli presented in the lower-central visual field at (0° azimuth, −10° elevation). The vertical line indicates the spatial frequency threshold, estimated by fitting a logistic function. **c** Spatial frequency thresholds for four mice at the three locations (data from the four lateral locations were similar and is pooled). Spatial frequency thresholds were significantly higher for the focea than the inferior location for all four mice (all *p* < 0.01, likelihood ratio test, two-tailed).

Does this reduced single-cell RF size in the focea result in higher spatial resolution vision? To test this hypothesis, we measured the visual acuity of four mice at different locations in their visual field. We trained mice on a go/no-go visual detection task (Fig. 7a) and presented 30° diameter circular sinusoidal grating stimuli of different spatial frequencies (between 0.25 and 0.75 cycs/deg) at six different spatial locations (focea [azi = 0°, ele = +20°], the inferior-central field [azi = 0°, ele = −10°] and at four lateral locations [azi = ±35°, ele = +20°/−10°]). The performance of the mice, as measured using d-prime (see Methods), decreased with increasing spatial frequency and we fit the data with a logistic function (Fig. 7b) taking the inflexion point of the curve as the threshold spatial frequency. Spatial frequency thresholds ranged from 0.4 to 0.65 cycs/deg, a range similar to that measured previously using a variety of behavioral techniques^17–19^. There were significant differences in spatial frequency threshold across spatial location in all four mice (Supplementary Fig. 7, likelihood ratio test, see Methods, all *p* < 0.01). We focused on the comparison of the focea to the inferior-central position because they were both in the binocular zone and to ensure that our results could not be explained by differences in viewing angle. The acuity was higher at the focea (mean threshold = 0.56 cycs/deg) than at the inferior-central position (mean threshold = 0.47 cycs/deg). Foceal acuity was also higher than at the lateral locations in three out of four mice (Fig. 7c), but we interpret this result with caution due to differences in binocularity and viewing angle. The higher spatial acuity at the focea suggests a functional consequence of the decreased scatter and the smaller RFs.

### Eye movements in freely moving mice hold the focea in front of the animal

**Fig. 8:**
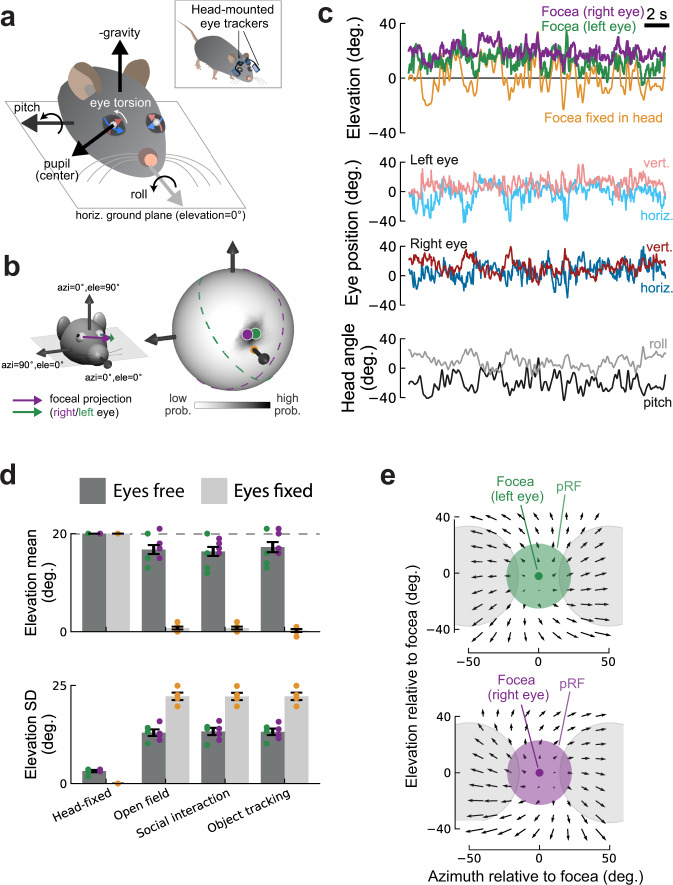
Compensatory eye movements in freely moving mice keep the focea ahead of the animal. **a** Tracking of head tilt (pitch and roll) and left and right eye positions in a freely moving mouse (inset). Illustration of head pitch and roll axes (relative to the ground), eye torsion (white arrow), and pupil centers (white dots) in angular eye coordinates (blue and red arrows). **b** The spherical coordinate system used in this study with [azi = 0°, ele = 0°] pointing toward the animal’s nose. The arrows indicate the foceal projection of the right (purple) and left (green) eye. Distribution of angular foceal projections in the same reference frame for an example mouse during spontaneous locomotion in an open field environment (right). Light and dark shading indicate regions of low and high probability, respectively. Dots indicate circular median position of the focea for the left (green) and right (purple) eye. Dashed lines indicate monocular visual fields for the left and right eyes (same color schema; 180° visual collection angle for each eye) showing that the focea fell into the binocular zone. **c** Thirty-second segment showing elevation of left/right foceas (top), horizontal, and vertical eye position of left and right eye (middle) and head pitch and roll (bottom) for the example mouse in (**b**). Same conventions as in (**a**). Control condition (“Focea fixed in head”) in top panel shows what the elevation of the focea would have been in the absence of stabilizing eye movements. **d** Average circular mean elevation (top) and standard deviation (SD, bottom) of the left/right foceas for the same mice either head-fixed or in three different head-free contexts (open field, social interaction, object tracking). In all three contexts, eye movements counteracted head movements to stabilize the elevation of the focea relative to the ground. Same color schema as in panels **a**–**c**. Dark gray bars show means for the focea and light gray bars show means for the control condition (“Focea fixed in head” in panels **a**–**c**). Means computed across eyes and mice. Data from four mice. Mean ± SEM. **e** Optical flow field during locomotion (body speed >10 cm/s) for the left (top) and right (bottom) eye. Optical flow vectors were computed for discrete grid points (spacing 10°) centered at the focea using head and eye positions and the geometry of the environment. Black arrows show average flow vectors across four mice. Green/purple shaded circles illustrate pRF size at the focea. Gray circles, pRF size at an azimuth of 50°.

Next we investigated whether mice take advantage of the higher spatial resolution of the focea by positioning it at strategic locations when they are free to move. We used a recently developed system to track the head and the horizontal and vertical eye movements in freely moving mice^20^ (Fig. 8a, inset). To also estimate eye torsion (rotation around the gaze axis) in freely moving animals, we took advantage of the stable relationship between head tilt and eye torsion, which we measured in head-fixed animals (Fig. 8a and Supplementary Fig. 8a; also see Methods). Importantly, the eyes of mice are positioned laterally with a lateral direction of gaze (defined as the ray going from the center of the eye through the center of the pupil). Their focea is therefore not aligned with the gaze direction, unlike in primates where the fovea is at the center of gaze. Our head-fixed experiments revealed the focea as the region of cortex representing the direction [azi = 0°, ele = 20°] in a spherical coordinate system in which azimuth is measured relative to the animal’s nose and elevation relative to the horizontal plane (Fig. 8a, b). In a new set of mice we first determined the location of the focea in eye coordinates, i.e., as the angle between [azi = 0°, ele = 20°] and the average position of the pupil, while they were head-fixed. We combined this angle with the eye and head position when the animals were freely moving, to determine the direction represented by the focea, with azimuth relative to the mouse’s nose and elevation relative to the horizontal ground plane (Fig. 8b, left panel). We refer to this direction as the “foceal projection.” During natural behaviors, mice move their eyes and head and the foceal projection could potentially point to different locations. To determine the probability distribution of the foceal projection we first projected it to a sphere surrounding the animal’s head during natural behaviors (Fig. 8b).

The azimuth position of the foceal projection was largely confined to azimuths close to zero and the foceal projection fell in the binocular zone of freely moving mice 99.5% of the time (Fig. 8b). Mice make strong compensatory eye movements to stabilize their gaze close to the horizon^21^. We found that these eye movements, together with eye torsion, held the foceal projections at an elevation of approximately 10°–20° above the horizontal plane (Fig. 8b, d and Supplementary Fig. 8b), even during strong changes in head pitch or roll (Fig. 8c). This was true across a range of typical mouse behaviors such as locomotion through an open field, social interactions, and an object-tracking task. It was also consistent across mice (Fig. 8d and Supplementary Fig. 8b). To test if eye movements were critical in stabilizing the elevation of the foceal projection, we re-estimated its position had there been no compensatory eye movements (i.e., we assumed that the eyes were fixed in the animal’s head—yellow trace in Fig. 8c). The fixed foceal projection had a different position than the unfixed projection in all three behavior types (paired *t*-test, all *p* < 0.001). It often pointed toward the ground (Fig. 8b–d) and its variation was considerably larger than if the compensatory eye movements were taken into account (Fig. 8d) (paired *t*-test, *p* < 0.001 for all three behavior types). In conclusion, freely moving mice keep the foceal projection at a relatively stable position in front of them. This position aligns well with the direction of the foceal projection in head-fixed animals.

We hypothesized that pointing the focea ahead might be of strategic importance, especially during locomotion, because it would position it at the “focus of expansion” (FOE) of the optic flow field during forward motion^22^. The FOE is the point in the optic flow field from which all visual motion seems to emanate. Furthermore, the relatively stable visual input close to the FOE may be critical for object identification and navigation during locomotion^23^. To test if the higher resolution focea is close to the FOE in freely moving mice, we identified periods dominated by forward locomotion (body speed >10 cm/s) during a visual object-tracking task. The task involved approaching a visual object on a computer screen on one side of an experiment chamber, tracking the object and collecting a reward on the other side of the chamber (Supplementary Fig. 8c). We used a model of the environment to compute the optical flow for a grid of retinal locations (grid spacing 10°) relative to the focea (see Methods). The resulting optical flow patterns were approximately radial and consistent across mice (Supplementary Fig. 8e). The FOE was close to the foceal projection of both eyes (Fig. 8e) and the magnitude of optic flow increased with r-eccentricity in all four mice (*p* < 0.001, Wald test). This suggests that a potential role of the higher resolution focea could be processing of the more stable visual patterns close to the FOE.

## Discussion

Here we have demonstrated a hitherto unknown similarity in the organization of pRF sizes in V1 and in the RF sizes of single neurons in higher visual areas between mice and primates^10^, in spite of other, important differences between species. In primates, the RFs of individual V1 cells are smallest near the fovea^15^, which reflects the homogeneous sampling of a steep gradient in the density of rods and cones in the retina. In mice, the distribution of ganglion cells is more uniform with only a four-fold difference between the highest density regions (in central-temporal parts of the retina) and lowest-density regions (in dorsal parts of the retina that receive light from below)^8^. There are functional differences between ganglion cells at different positions in the retina^24–26^ but we did not observe a strong relationship between eccentricity and the size of RFs of individual mouse V1 neurons, with the possible exception of larger RFs at very negative azimuths. Instead, a gradient was evident in the scatter of RFs of cell bodies and neuropil. While mouse visual cortex is broadly retinotopically organized, there is considerable scatter of RF positions at a local spatial scale^14^. The origin of this scatter is unknown but it could be due to scatter in the arrangement of feedforward axonal projections from the LGN and in the projections of retinal ganglion cells to the LGN. When considering an aggregate RF over several cells, regions with higher scatter will contain larger pRFs. Our results indicate that this scatter is systematically organized across mouse visual cortex, with lower scatter, and hence smaller pRFs, in the focea.

We also observed an increase in CMF within the binocular region of V1, with CMF values being two to three times higher close to the vertical meridian than in the monocular regions of cortex, in agreement with previous studies^3,4,9^. This over-representation of binocular regions of space could reflect a requirement for cells representing multiple retinal disparities in this region of space^27–29^. The absence of a dependency of CMF on elevation is in line with this view. We considered the possibility that binocularity decreased scatter and thereby contributed to the smaller pRFs at the focea. If RF scattering had a monocular origin and is independent for the two eyes, pooling across the eyes could reduce scatter. However, we observed that binocularity did not explain the variations in RF scatter and that it was a poor predictor of pRF size. Taken together, our results reveal several factors that could work together to produce a higher visual resolution in the focea: a reduction in RF scatter, an increase in the number of cells representing binocular regions of space, and a decrease in the RF size of individual neurons in a subset of the higher visual areas.

The relationship between r-eccentricity and pRF size in V1 was steeper when measured using wide-field imaging than using the scatter of the RFs of cell bodies and neuropil measured with two-photon imaging. This difference is likely due to a combination of factors. The signal from a single wide-field pixel comprises light scattered from different depths, whereas the two-photon signals measured here were largely confined to a single depth plane in layer 2. Furthermore, wide-field images may be dominated by neuropil signals. Neuropil contains neural processes from multiple cells and, in addition, reflects a contribution from feedback axons, which have larger RFs than V1 neurons, terminate in large numbers in the upper layers of cortex^30–32^, and convey substantial inputs from visual field regions outside the local V1 RFs^33^. An intriguing possibility is that feedback to the foceal representation may itself be more targeted than feedback to peripheral regions, and that this may contribute to the smaller pRFs observed in this region, although further experiments are needed to probe this hypothesis.

For the decreased scatter at the focea to be of functional significance, it should be converted into smaller RFs of cells in downstream areas. The sampling strategy of cells in mouse higher visual areas is not completely understood, but if cells in higher areas sample from equally sized V1 regions, as is the case in cats and primates^34^, they will have smaller RFs at the focea than in the periphery (Fig. 6a). We indeed observed smaller cellular RFs at the focea in areas LM and AL, but not in RL, AM, and PM, giving insight into the functional specializations of these areas. The smaller foceal RFs could be a result of integrating over less scattered representations in V1 but could also be generated de novo in LM and AL, which receive considerable direct input from the LGN^35,36^. The weaker relationship in areas RL, AM and PM may stem their small representation of the focea because RFs in RL and AM are located predominantly in the inferior visual field and PM represents more temporal locations^3,37,38^. These areas may be required for visual tasks which do not require the higher resolution of the focea, such as monitoring movement in the periphery (PM^39^) and visuotactile integration of visual inputs close to the whiskers (RL^28^).

The smaller foceal RFs of higher visual areas were associated with a higher acuity. Previous studies demonstrated that mice are faster and more accurate in detecting low-contrast visual stimuli in the binocular region of space compared to monocular regions^40^ and our behavioral data extend these findings. The acuity was higher at the focea than in the lower-central field (Fig. 7b, c), a difference that cannot be explained by differences in CMF or binocularity. Hence, like primates, mice have a region of improved visual resolution that they may use for more detailed visual analysis.

Primates make rapid and frequent eye movements to fixate upon items of interest in the visual scene, utilizing the higher resolution at the fovea for a more detailed analysis^41,42^. More cognitive aspects of vision, such as visuospatial attention, are closely related to the control of eye movements^43–45^. Freely moving mice make saccades that are tightly locked to head movements^20^. The combined effects of eye and head movements produce a “saccade-and-fixate” sampling of the visual scene that is reminiscent of that in primates^21,46^, although mice make saccades that are small relative to the size of the visual scene^47^. In addition to saccades, mice make slower gaze-stabilizing eye movements that are coupled to changes in head pitch and roll^21,48^. Our results show that when freely moving mice orient their head during visual exploration, they move their eyes to compensate for head movements to help keep the focea at a location ahead of the animal at an elevation of approximately 20°. As freely moving mice make head and eye movements that shift gaze predominantly parallel to the ground, this suggests that mice use the focea to scan around the horizon, potentially helping the animal to identify safe locations or behaviorally relevant objects such as insects. Indeed, during hunting mice keep prey in a visual field region that is close to zero azimuth^46,49–51^ orienting their head toward the prey animal and shifting their eyes to recenter their gaze^46^. A further potential role of the enhanced spatial resolution ahead of the animal could be to gauge optical flow to provide information about the mouse’s heading direction and the relative distance to objects in the environment. As near points move fast and far points move slowly such a mechanism could improve processing in the “FOE” of the optical flow field during locomotion.

In combination with previous work, our study provides important insights into cortical organization at a more global scale^52–54^, revealing organizational principles that are not apparent at the local level. For example, a recent study demonstrated that mouse visual cortex contains a global map of orientation preference^52^, which is not observable at the spatial scales obtained in a typical field-of-view of a two-photon microscope. A picture of the mouse cortical visual system is emerging that mirrors many of the organizational principles in primates, albeit at a coarser scale.

## Methods

### Animals

All experimental procedures complied with the National Institutes of Health Guide for Care and Use of Laboratory Animals and the protocol was approved by the animal ethical committee of the Royal Netherlands Academy of Arts and Sciences and the Centrale Commissie Dierproven; all experiments were performed in accordance with the relevant guidelines and regulations. For wide-field imaging we used 28 Thy1-GCaMP6f mice^12^ aged between 2 and 14 months. Eleven mice (3 female) were imaged bilaterally and were held on a reverse day/night cycle during the entire experiment. Seventeen mice (14 female) were imaged unilaterally with a normal day/night cycle. These 17 mice were also used for the electrophysiological experiments plus an additional 11 animals that were not imaged yielding a total of 28 animals. For two-photon imaging we used three extra Thy1-GCaMP6f to tile area V1 and five Thy1-GCaMP6f mice to image the higher visual areas (these mice were held on a normal day/night cycle). All mice were housed in a facility with a temperature range of 21–24 °C and relative humidity of 40–60%. Four C57Bl/6J mice (Charles River, aged 44–49 days) were used for head- and eye-tracking experiments in freely moving mice. The experimental procedures for head- and eye-tracking experiments were carried out in accordance with a UK Home Office Project License, approved under the United Kingdom Animals (Scientific Procedures) Act of 1986.

### Clear skull surgery for wide-field imaging

To visualize the surface of the cortex we used the “clear skull” technique in which the natural transparency of the mouse skull is made permanent through the application of cyanoacrylate glue and an optically transparent cement. Starting a week before surgery, mice were handled 5–10 min per day. On the day of surgery, anesthesia was induced using 3–5% isoflurane in an induction box and then maintained using 1.2–2.5% isoflurane in an oxygen enriched air mixture. Then, 5 mg/kg Metacam in saline (0.5 mg/ml) was subcutaneously injected as a general analgesic to prevent pain and aid in the recovery of the animals. Mice were mounted on a stereotactic frame to allow precise localization of target areas and stable working conditions. Depth of anesthesia was monitored by frequently checking paw reflexes and breathing rate. During the entire procedure the temperature of the animal was monitored and kept between 36.5 and 37.5 °C, using a heating pad that received feedback from a rectal thermometer. The eyes were covered with ointment to prevent dehydration. The area of incision was shaved, cleaned, and lidocaine spray was applied to the skin as a local analgesic. An incision in the skin was made along the anteroposterior midline, and the skin was gently pulled laterally, exposing the area of the skull above the cortex and the area posterior to lambda. The bone of the target area was cleaned by removing remaining tissue and briefly applying H_2_O_2_. After carefully drying the area, a thin layer of adhesive (Kerr Optibond or cyanoacrylate glue (Bison)) was applied to the bone, thereby making the bone transparent. This effect occurs over the course of the following 2 days and is referred to as “clear skull cap” technique. A platform of dental cement (Heraeus Charisma) was built to place the head-bar. Multiple layers of cement were used to secure the head-bar on the skull. For mice that were imaged bilaterally, a thin layer of clear dental cement (C&B super-bond), and nail polish were applied (Electron Microscopy Sciences), to reduce light glare. On the outer edges of the imaging area a small wall of cement (Heraeus Charisma) was added to keep the skin from retracting over the area of interest. In mice that were unilaterally implanted and used for electrophysiological experiments, stainless steel screw(s) were implanted in the skull for referencing and grounding. The animal was monitored and kept warm while waking up, and had a minimum of 2 days to recover before acclimatization to the recording setup.

### Cranial window implantation for two-photon imaging

For two-photon imaging of V1, three mice underwent surgery to implant a head-ring for immobilization and a cranial window to allow imaging of activity in the brain. Animals were anesthetized as above and the area of skull above right visual cortex was exposed. The skull was cleaned by removing any remaining tissue with blunt dissection and briefly applying H_2_O_2_ after which dental primer was applied (Kerr Optibond). A circular metal ring was fixed on the skull with light cured dental cement, centered on the visual areas of the right hemisphere and parallel to the plane of imaging. A circular craniotomy of diameter 5 mm was etched out on the bone, centered on 0.5 mm anterior to lambda and 2.5 mm lateral from the midline. After carefully thinning the bone along the outer diameter of the craniotomy, the bone flap was slowly lifted without damaging the dura. Once exposed, the dura was constantly kept moist with warm ACSF or saline. The craniotomy was closed with a double-layered glass coverslip, with the outer glass resting on the skull. The glass coverslip (cranial window) was fixed using dental cement (Vivadent Tetric Evoflow). The animal received painkillers (5 mg/kg Metacam) and a recovery period after the surgery as described above.

For animals in which we imaged the higher visual areas we first implanted a head-ring for head-fixation as described above. After 2 weeks of recovery we mapped pRFs to allow recognition of the different higher visual areas (as described above). We then injected each animal with AAV1-CaMKII-GCaMP6f-WPRE-SV40 (Penn Vector Core, University of Pennsylvania, USA) in V1 (100 nl) and LM, AL, RL, AM, PM (50 nl each) at an injection speed of 20 nl/min distributed across two depths (400 µm, 200 µm below the pial surface) to enhance the GCaMP signal. In the same surgery we performed a craniotomy and implanted a cranial window as described above.

### Wide-field imaging

Mice were placed under a wide-field fluorescence macroscope (Axio Zoom.V16 Zeiss/Caenotec-Prof. Ralf Schnabel), which allows imaging of a large part of the cortical surface. The head-bar of the mouse was positioned so that the nose of the mouse was located at the horizontal center of the LCD screen (zero azimuth), in the vertical plane the head of the mouse was leveled along the anterior-posterior axis holding it perpendicular to the screen and the position of the nose was taken as the point of zero elevation. Images were captured at 20 Hz by a high-speed sCMOS camera (pco.edge 5.5) and recorded using the Encephalos software package (Caenotec-Prof. Ralf Schnabel). The size and position of the right pupil was tracked at either 50 or 100 Hz using custom-built software. Movement of the mouse was monitored using a piezo plate under the front paws of the mouse that was sampled at 50 or 100 Hz. Removal of trials in which the animal blinked or moved above a pre-defined threshold had very little effect on the quality of the maps and so all trials were included. Visual stimuli were created using COGENT graphics (developed by John Romaya at the LON at the Wellcome Department of Imaging Neuroscience) running in MATLAB. Stimuli were presented on a 122 × 68 cm LCD screen (Iiyama LE5564S-B1) at a resolution of 1920 × 1280 pixels running at refresh rate of 60 Hz. The viewing distance was 14 cm, yielding a field-of-view of 77° × 43°. The stimulus was constructed from a static checkerboard pattern composed of black (0 cd/m^2^) and white (40 cd/m^2^) checks of 5 × 5 visual degrees. To create the mapping stimuli, the checkerboard was visible through a bar-shaped aperture and the rest of the screen remained gray. The bar was 20° in width and could be angled at an orientation of 0°, 45°, 90°, or 135°. For each orientation, the bar was presented at different positions, tiling the entire screen. To ensure that the size of the check in degrees of visual angle was constant with eccentricity, we corrected for the variable viewing distances inherent in the use of a flat-screen monitor using a previously described spherical correction technique^2^. We masked out regions of the screen that were more eccentric than ±70° with a black mask and removed bars outside unmasked regions, yielding a total of 31 bar stimuli. We also carried out sessions in which we used smaller bars (10° in width) yielding a total of 58 bars. The results were similar to those using 31 bars and are combined here. The bar stimuli and mask were presented for 500 ms, followed by and inter-stimulus interval consisting of only the mask filled with mean gray level (20 cd/m^2^) for 3.6 s. Each stimulus was presented 15 times and the total duration of a mapping experiment was 31.8 min.

### Wide-field signal processing

The raw 16-bit images were downsampled from 1600 × 1600 pixels to 800 × 800 pixels by averaging the signals across squares of 2 × 2 pixels. Each image was realigned with the first image in the sequence using a rigid-body transformation (i.e., only translation and rotation). Each image was then smoothed using a 7 × 7 pixel moving window (which corresponds to approximately 85 × 85 μm), replacing each pixel-value with the mean of the neighboring three pixels in the *x* and *y* directions. For each presentation *i* of stimulus *s* we took the baseline fluorescence *F0*_*s,i*_ as the mean fluorescence between −0.25 and 0 s and the stimulus response *F*_*s,i*_ as the mean fluorescence between 0.15 and 0.4 s. The mean evoked response for stimulus *s*, *E*_*s*_, was then calculated as:

1 $$\begin{array}{c}{E}_{s}=\frac{{\sum }_{i=1}^{n}(({F}_{s,i}-F{0}_{s,i})/F{0}_{s,i})}{n}\end{array}$$

### Wide-field pRF analysis

The parameters of the best-fitting pRF for each pixel were estimated using a linear model. The pRF is assumed to take the form of a two-dimensional Gaussian envelop of the form:

2 $$\begin{array}{c}G({a}_{0},{e}_{0},\sigma ,\,a,\,e) \sim {e}^{-\frac{{(a-{a}_{0})}^{2}+{(e-{e}_{0})}^{2}}{2{\sigma }^{2}}}\end{array}\,$$

The three free parameters were *a*_*0*_ and *e*_*0*_: the center of the Gaussian in the azimuth and elevation directions and *σ*, the standard deviation of the Gaussian, which we converted to FWHM using the equation:

3 $$\begin{array}{c}{\rm{FWHM}}=2\sqrt{2{\rm{ln2}}}\sigma =2.35\sigma \end{array}$$

Gaussians were constructed with center coordinates (*a*_*0*_, *e*_*0*_) ranging from −90° to +90° of azimuth and −60° to +60° of elevation with a spacing of 2° of visual angle. The FWHM ranged from 20° to 120° in steps of 2°. Gaussians with centers that lay outside the stimulated region of space were removed. This yielded a total of 376,431 Gaussians.

To make a predicted set of responses, we multiplied each Gaussian on a point-by-point basis with a model of each bar stimulus. The dark- and light-checks of the bar stimulus are assumed to contribute equally to the GCaMP response and the aperture of the stimulus was used: the stimulus strength *S*(*a*,*e*,*i*) was 1 within the aperture and 0 outside. The predicted response *R*(*i*) to stimulus *i* is proportional to:

4 $$\begin{array}{c}R(i) \sim \mathop{\sum}\limits_{a}\mathop{\sum}\limits_{e}S(a,e,i)G({a}_{0},{e}_{0},\sigma ,a,e)\end{array}$$

The predicted response is related to the observed response of each pixel via an unknown gain parameter *β*_*g*_. To estimate *β*_*g*_ we assume the observed response (*y*) of each imaged pixel to stimulus *i* is given by:

5 $$\begin{array}{c}y(i)={\beta }_{g}R(i)+\varepsilon \end{array}$$

where *ε* is a normally distributed error term. We estimated *β*_*g*_ using linear regression.

The goodness-of-fit of the model was assessed using the sum-of-squares difference between the observed and predicted responses and the Gaussian minimizing this error term was taken as the pRF for each pixel. To reduce calculation time, we estimated the best-fitting Gaussian for every other pixel in the image and used linear interpolation to determine the parameters of the remaining pixels. This results in maps of azimuth, elevation, and pRF size (FWHM). These maps were thresholded by Pearson’s correlation between the model and the data (threshold = 0.75). To analyze the shape of the relationship between pRF location and size we fit a linear model to the azimuth and elevation data recentered on the focea of the form:

6 $$\begin{array}{c}{\rm{pRF}}_{\rm{FWHM}}=G.ecc+c\end{array}$$

7 $$\begin{array}{c}{r}_{{\rm{ecc}}}={\rm{arctan}}(\sqrt{{(\tan (\varDelta a))}^{2}+\frac{{(\tan (\varDelta e))}^{2}}{{(\cos (\varDelta a))}^{2}}})\end{array}$$

where *ecc* is the spherical angle between two points on a sphere^3^, Δa is the difference in azimuth of the pRF center from 0°, and Δe is the difference in elevation from 20°. In other words the pRF size was assumed to be linearly related to the spherical distance of the pRF center from the focea. *G* and *c* were estimated using nonlinear minimization (fminsearch.m in MATLAB) minimizing the sum-of-square errors weighted by the reciprocal of eccentricity to lessen the impact of outliers at large eccentricities.

### Field-sign analysis

Visual areas were identified using field-sign analysis as reported previously^3^. Briefly, azimuth and elevation maps were downsampled to a resolution of 40 pix/mm^2^ using nearest neighbor interpolation and smoothed with a 7 × 7 pixel wide sliding window. The direction of the gradient in the azimuth and elevation maps were calculated using a Sobel operator (“imgradient.m” in MATLAB) and the resulting images were converted to field-sign using the following equation:

8 $$\begin{array}{c}{\rm{FieldSign}}={\rm{sin}}(\overrightarrow{Ele}-\overrightarrow{Azi})\end{array}$$

where the arrowed variables indicate the direction of the gradient in elevation and azimuth maps in radians. Pixels with poor pRF fits (Pearson’s *r* < 0.75) were set to NaN. The resulting map was smoothed with a 13 × 13 pixel sliding window and thresholded at a particular value (*t*) using the following logic:

9 $$\begin{array}{c}\left\{\begin{array}{l}\hskip-1.5pc{\rm{value}} \,> \, t;{\rm{FieldSign}}=1\\ {\rm{value}} \,< -t;{\rm{FieldSign}}=-1\\ -t \,<\, {\rm{value}} \,<\, t;{\rm{FieldSign}}=0\end{array}\right.\end{array}$$

Here we used a value of *t* = 0.3. The thresholded maps were processed using morphological operators as described in Garrett et al. to produce contiguous regions with non-overlapping representations of space. V1 was always easy to identify as the largest region with a negative field sign. Higher visual areas were identified by their location relative to V1. To produce an average map of visual areas (Fig. 1e) we first recentered the individual maps on the center of V1 and resized them by dividing the major and minor axes of V1, given the variation in the shape, size, and location of mouse visual areas^55^. We then aligned the average map to those published in ref. ^3^.

### Electrophysiology

Electrophysiological recordings were carried out in 17 mice that were imaged unilaterally under the wide-field microscope and 11 mice that were not previously imaged. Electrode penetrations were targeted to V1 using the pRF maps obtained using wide-field imaging or stereotactic coordinates. Three weeks after the initial surgery (described above) a craniotomy was made over V1 under general anesthesia and appropriate analgesia (as described above) at least 24 h prior to the recording session and was sealed with Kwik-Cast (WPI). During electrophysiological recording sessions the mice were placed on a treadmill, head-fixed and allowed to run or sit freely. We inserted a linear-array recording electrode (A1×32–5mm-25-177, NeuroNexus, 32 channel probe, 25 micron spacing) in V1 and lowered it to around 1 mm below the brain surface and adjusted the depth of the electrode with reference to the current source density profile as reported previously^56^ to ensure coverage of all layers. We amplified the electrical signal from the electrodes and sampled it at 24.4 kHz using a Tucker–Davis Technologies recording system. We removed muscle artifacts by re-referencing each channel to the average of all other channels before filtering the signal between 500 and 5000 Hz. We detected spikes by thresholding (positive and negative threshold) the band-passed signal at four times an estimate of the median absolute deviation and convolved the detected spikes with a Gaussian with a standard deviation of 1.3 ms and an integral of 1 to derive an estimate of multi-unit spike rate.

We measured the RF of the units recorded at each electrode using a sparse-noise stimulus. Visual stimuli were projected onto a back-projection screen placed 15 cm from the mouse using a gamma-corrected PLUS U2-X1130 DLP projector (mean luminance = 40.6 cd/m^2^). The size of the projection was 76 × 56 cm yielding a field-of-view of 136° × 101.6° with a pixel resolution of 1024 × 768 and a refresh rate of 60 Hz. The sparse-noise stimulus consisted of four white checks (8° by 8°, 40 cd/m^2^) on a black background presented for 250 ms with a 250 ms inter-trial interval. The checks (>30 presentations per check) were positioned on a grid ranging from −64° to 16° horizontally and −22° to 66° vertically relative to the mouse’s nose with negative values indicating the right hemifield and corrected for flat-screen distortion as described above. We averaged the MUA response evoked by each check in a time window from 50 to 400 ms after stimulus onset to obtain a map of visual responsiveness and fit a 2D Gaussian to estimate the width and center of the RF. The quality of the fit was assessed using *r*^2^ (using 0.33 as the cut-off) and a bootstrapped variability index (BVI), which estimated the reliability of the estimate of the RF center. We resampled an equal number of trials as in the original dataset (with replacement) and regenerated the Gaussian fit. The BVI is the ratio of the standard deviation of the RF center position and the standard deviation of the fitted Gaussian. RF estimates of recording sites with a BVI larger than 1 were considered unreliable and removed from the analysis.

### Two-photon imaging

Two weeks after the implantation of the imaging window, we started to habituate the mice to head immobilization while they could run on a running belt under the two-photon microscope (Neurolabware). We imaged frames (764 × 480 pixels, which covered 1 × 0.1 mm) at 15.7 Hz through a 16× water immersion objective (Nikon, NA 0.80) at 1.7× zoom at a depth of 120–300 μm. We either targeted V1 or a higher visual area based on the pRF maps obtained under wide-field imaging as described above. A Ti-Sapphire pulsed laser (MaiTai, Spectra Physics) was tuned to 920 nm for delivering excitation light. We mapped the RF locations of the neurons using a sparse-noise stimulus. We presented 12° × 12° white (38 cd/m^2^) squares on a black background (0.05 cd/m^2^) on a grid ranging from −78° to 18° horizontally and –21° to 51° vertically relative to the mouse’s nose. On each trial, four non-adjacent squares were displayed for 250 ms, which was followed by a delay of 500 ms. The squares were presented 20 times at each location in the grid. We used CAIMAN^57^ for pre-processing of the recorded signal. We performed rigid motion correction for small shifts in the data due to motion of the animal, followed by the extraction of regions-of-interest (ROIs) and the ΔF/F. ROI components were identified using a constrained non-negative matrix factorization algorithm^57^ and were further classified into cells and neuropil regions using a pre-trained convolutional neural network based classifier, from the CAIMAN matlab github library: https://github.com/flatironinstitute/CaImAn-MATLAB/wiki/Component-classification-with-a-convolutional-neural-network. Components classified as cells were used for the single-cell analysis. We calculated RFs based on the difference in mean response in a window after stimulus onset (2–8 imaging frames; 126–504 ms after stimulus onset) compared to a baseline window (−5 to −1 frames: −315 to −63 ms) in response to each square. We fit a linear regression model to estimate the average responses to the squares of the grid, regressing out the influence of running and the interaction between the visual stimulus and running. We fit a circular 2D Gaussian to the beta weights for every grid location to estimate the RF center and its full width at half maximum (see above for details). We evaluated the quality of the fit using the *r*^2^ value and the BVI (see above; *r*^2^ of the Gaussian fit >0.33, BVI <1, and a positive visual response).

### Model of V1

To conceptualize the relationship between CMF and RF scatter we generated models of V1 using parameters measured in the two-photon experiments. Each model contained 400 cells positioned on a 20 × 20 regular grid spanning 2.4 mm in the *x* and *y* directions. We assumed a foceal CMF of 0.02 mm/deg. Model A simulated a relationship between eccentricity and CMF. The eccentricity of a cell’s RF was determined entirely by the position of its cell body in cortex, as given by:

10 $$ecc(n)=\frac{1}{0.02}z{(n)}^{a},\,{\rm{where}}\,z(n)=\sqrt{x{(n)}^{2}+y{(n)}^{2}}$$

where *x*(*n*) and *y*(*n*) were the position of the *n*th cell body in the azimuth and elevation encoding directions in cortex in mm. The exponent *a* controlled the steepness of the relationship between CMF and eccentricity and was set to 1.5 in the example in Fig. 3. The azimuth and elevation positions of the cell’s RFs were then generated as:

11 $$\begin{array}{c}\begin{array}{c}azi(n)=ecc(n).{\rm{cos}}\Big({\rm{arctan}}\Big(\frac{y(n)}{x(n)}\Big)\Big)\\ ele(n)=ecc(n).{\rm{sin}}\Big({\rm{arctan}}\Big(\frac{y(n)}{x(n)}\Big)\Big)\end{array}\end{array}$$

In Model B, the same equations were used with the exception that cortical magnification was set to be constant by using a value of *a* = 1, and normally distributed noise was added to the eccentricity position. The standard deviation of the noise increased with eccentricity as follows:

12 $$\begin{array}{c}ecc(n)=ecc(n)+N(\mu ,\sigma ),\,{\rm{where}}\,\mu =0,\sigma =0.18.ecc(n)\end{array}$$

The value of 0.18 was estimated from the two-photon data.

### Measurement of cortical magnification factor and RF scatter

To estimate CMF in the azimuth and elevation directions we related RF positions of single V1 neurons to the position of their cell body in cortex. We first rotated the axes of the cortical image so that the representation of azimuth changed principally along the *x*-axis and the representation of elevation along the *y*-axis. We then estimated the location of the foceal representation by finding the point in cortex with a representation closest to [0° azimuth, 20° elevation]. This was done by moving 100 µm radius windows over the cortical surface and computing the mean eccentricity of cells falling within the window. We fit the relationship between the *x* (*y*) position of the cell bodies and the position of their RF in the azimuth (elevation) direction by fitting an exponential function using robust nonlinear least-absolute residual regression:

13 $$x={e}^{\frac{{\rm{log}}(\frac{v}{a})}{b}}$$

where *x* is the position of the cell body along the azimuth-encoding direction in millimeters and *v* is the azimuth of the cell’s RF in visual space and *a* and *b* are constants. To estimate CMF we evaluated the fitted function at azimuth values ranging from −60 to +20 in 5° steps and then took the difference between neighboring values. A similar procedure was followed for elevation, with evaluation points at −30 to +30 in 5° steps and eccentricity, with evaluation points at 0°–60° in 5° steps. To estimate the scatter of RFs we took the residual difference between each RF’s position and the fitted exponential function. The azimuth (elevation, eccentricity) data were ordered from negative to positive and we evaluated the interquartile range of the residuals in ten non-overlapping bins, each containing 10% of the cells. The mean azimuth (elevation, eccentricity) value of the cells in the bin was taken as the center point of the bin and a linear regression was used to estimate the slope of the relationship between the central azimuth of the bin (elevation, eccentricity) and interquartile range. The significance of the slope of the fit was assessed by a bootstrapping procedure. An identical number of cells were resampled with replacement and the exponential function was fitted to the data. Under the null hypothesis, the scatter of the residuals around the fit does not depend upon the azimuth (elevation, eccentricity). We therefore generated a null distribution of slope values by scrambling the order of the residuals and recalculating the interquartile range and regression slopes. Significance was assessed as the proportion of slope values from the null distribution that were greater than the observed value in the original dataset. We analyzed the relationship between binocularity and RF scatter in a similar fashion. Cells were divided into monocular (azimuth >±15°) and binocular (azimuth <±15°) groups. We then took the difference in standard deviation of the residuals from the exponential function described above between the monocular and binocular group. This difference should be positive if RF scatter is less in the binocular regions of cortex. This difference was compared to a null distribution of differences obtained by scrambling the order of the residuals. Significance was assessed as the proportion of differences that were greater than the difference obtained in the original dataset.

### Two-photon pRF analysis

To calculate the aggregate RF size from the two-photon images we first focused on the signals isolated from single cells as described above. We only included cells in which we could reliably measure RFs using the criteria described above. We centered analysis windows on each cell ROI identified in the image with radii of 50, 100, 200, and 400 μm. We then included the RF of each cell that fell within the analysis window. A circle with a diameter of the FWHM of the Gaussian fit of each RF was overlaid in space and the area of the convex-hull of the overlaid Gaussians was taken as an estimate of the pRF. The pRF diameter in degrees was then quantified using the following equation:

14 $$\begin{array}{c}{\rm{pRF}}_{{\rm{diam}}}=2\sqrt{\frac{{\rm{area}}}{\pi }}\end{array}$$

where area was the area of the convex hull in deg^2^. This pRF diameter estimate is based on the aggregate FWHM values of individual neurons and it is therefore equivalent to the FWHM of a circular Gaussian fitted to the wide-field data. We corrected for the fact that windows located toward the edge of V1 had less cells in them and therefore smaller pRFs by including the number of cells in the pRF, and the square-root of the number of cells, as co-regressors in the regression of pRF size on eccentricity.

To estimate pRFs from the raw images we first smoothed the raw images with a sliding mean smoothing window (77 × 77 pixels or approximately 100 × 100 μm) and then downsampled the image eight times. We then calculated the mean evoked response to each sparse-noise square from each pixel as described above. We fit Gaussian RFs to the resulting maps as described above to produce maps of azimuth, elevation, and RF size. The resulting maps from different two-photon images were stitched together, using linear interpolation to estimate map values in regions of missing or overlapping data. To analyze scatter we used a similar approach as described above. We moved analysis windows over the stitched maps (50, 100, 200, 400 μm radius), only including windows in which at least 75% of the pixels in the window contained data to reduce edge artifacts. The pRF size was calculated in the same manner as described above for the single cells. The fraction of pixels within each window that included data was included as a co-regressor in the regression between pRF size and eccentricity to remove remaining edge artifacts.

### Visual detection experiment

We tested visual acuity at different locations of the visual field by training five mice on a go/no-go task visual detection task. The mice were held on a reverse day/night cycle and a fluid restriction protocol with a minimal intake of 0.025 ml/g, while their health was carefully monitored. Mice were head-fixed in front of a 24-inch LCD monitor (1920 × 1200 pixels, Dell U2412M), placed 11 cm in front of the eyes and a custom-made lick-spout was positioned in front of the animal. Licks were registered by measuring a change in capacitance on the lick-spout with an Arduino and custom-written software. We initially trained the mice on a simple version of the task using a full-screen sinusoidal grating (contrast = 50%, spatial frequency 0.08 cycs/deg). Trials were initiated if the mice withheld from licking for 2 s. On “go” trials the grating was presented for 1 s, whereas on no-go trials the screen remained at the mean luminance for 1 s. The inter-trial interval was 4 s with a random jitter of ±2 s and a timeout of 5 s was added if the mice licked during a no-go trial. Performance was assessed using d-prime (*d*’):

$$d{\prime} =Z(HR)-Z(FAR),$$

15 $${\rm{in}}\,{\rm{which}}\,HR=\,\frac{nHits}{nHits+nMisses}\,{\rm{and}}\,FAR=\,\frac{nFalse\,Alarms}{nFalse\,Alarms+nCorrect\,Rejections}\,$$

and *Z* is the *Z*-transform. Once *d*’ reached an average value of 1.5 for these easily detectable stimuli we began presenting mice with gratings through circular apertures of 30° diameter centered at one of six different spatial locations (Fig. 5c: focea, defined as the point directly in front of the mouse at an elevation of 20°, i.e., (azi = 0°, ele = +20°), the inferior-central field (azi = 0°, ele = −10°), and at four lateral locations (azi = ±35°, ele = +20°/−10°). We varied the spatial frequency between 0.25 and 0.75 cycs/deg in steps of 0.1 cycs/deg. One mouse was excluded from the experiment as it was unable to perform above 70% hit-rate even at low spatial frequencies and increased contrast (70%). The remaining four mice were able to perform the task and, as expected, *d*’ decreased with increasing spatial frequency. We fit a logistic function to each mouse’s hit-rate by maximum likelihood using the Palamedes Toolbox in MATLAB. We constrained guess rates to be the false-alarm rate and lapse rate (i.e., 1 –maximum of the curve) to be the same for each spatial position. The inflexion point and slope of the function were free parameters that could vary per position. The spatial frequency threshold was determined as the inflexion point of the logistic function for each of the locations and each of the mice. To test whether the position of the stimulus had an effect on the inflexion point of the curve, we refit the data constraining the slopes to be the same for all positions, but allowing the inflexion point to vary (full model) and compared this to a restricted model in which the inflexion points were constrained to be the same for each position. The fits of the full and restricted model were compared using a likelihood ratio test:

16 $$\begin{array}{c}LR=-2({\lambda }_{{\rm{restricted}}}-{\lambda }_{{\rm{full}}})\end{array}$$

where *λ*_restricted_ was the log-likelihood of the data under the restricted model and *λ*_full_ was the log-likelihood under the full model. The value of LR was compared to a *χ*^2^ distribution with 1 degree of freedom to calculate the *p* value.

### Eye and head tracking in freely moving mice

Four male C57Bl/6J mice were implanted with a head-bar and three miniature connectors to attach two head-mounted cameras (one for each eye) and an inertial measurement unit (IMU) sensor as described in Meyer et al. (2020). The cameras measure the positions of the eyes as they rotate in the orbits while the IMU provides information about head tilt (pitch and roll). Mice were allowed to recover for at least 5 days and handled before the experiments began. In each mouse, we performed experiments in four different conditions: (1) spontaneous locomotion in a circular or rectangular open field environment (“open field,” 43 recordings, 10 min each), (2) social interaction with a second male mouse without head-mounted system (“social interaction,” 10 recordings, 10 min each), (3) performance of an object-tracking task where animals pressed and tracked a rectangle appearing on an IR touchscreen (“object tracking,” 38 recordings, recording duration 322 ± 129 s), and (4) head-fixation as in the neural recording experiments (“Head-fixed,” 29 recordings, 10 min each). See Meyer at al. (2020) for detailed description of these four conditions.

For each eye, horizontal and vertical angular eye positions (defined as the center of the pupil) were extracted from camera images and transformed into an eye, head, or spatial reference frame as described in ref. ^21^. Briefly, eye positions were extracted using a deep convolutional network (DeepLabCut) trained via transfer learning^58^ and transformed into angular horizontal and vertical eye positions (relative to the axis of the eye in a head reference frame). A geometric model of the position of the eye axes in the head was used to relate eye positions to the head with the position of the left or right eye axes at ±60° azimuth (relative to midline) and 30° elevation^59^. Head tilt relative to the horizontal ground plane was measured using the head-mounted IMU: pitch measures nose up or down whereas roll measures sideward head tilt.

To determine the position of the focea in eye coordinates, we first computed the eye positions corresponding to the focea (azi = 0°, ele = 20°) for each eye (left/right) using the inverse (i.e., transpose) of the 3D rotation matrix of the eye geometry model. For each mouse, the positions of the left/right foceas were computed using the average left/right eye positions measured during head-fixation. Thus, for a straight head as in the head-fixed recordings (pitch = 0° and roll = 0°) and the eyes in their average positions, the vectors indicating the directions of the foceas in space would point at azi = 0° and ele = 20° in a spherical coordinate system. In addition to horizontal and vertical eye movements, rodents also rotate the eyes around the resulting “gaze” axes (torsion) when pitching their heads up or down^60^. To also incorporate torsional eye rotations, we first estimated the relation between torsion and head pitch in two passively tilted mice by tracking features on the pupil circumference using a deep neural network^58^. While it is possible to track a small number of small-scale features on the pupil circumference in freely moving mice, these features were hard to identify when the pupil was small (e.g., due to bright light), occluded by the eyelid, when mice are making eye movements and when the pupil is large. Consistent with torsion measurements in freely moving rats^60^, we found that torsion in mice was approximately linearly related to head pitch with a value of around 0.325 (Supplementary Fig. 8a). This value was used in all analyses.

The distribution of left/right focea locations (Fig. 8b and Supplementary Fig. 8b) was computed using a grid of equally spaced points in spherical coordinates (spacing 1°). The radius of the sphere was 10 cm^27^ and the head of the animal was placed at the center of the sphere (nose pointing at ele = 0° and azi = 0°). For each tracked left or right angular eye position and corresponding pitch/roll values, we computed the direction of the focea in spherical coordinates and increased the count of the grid point closest to the vector of length 15 cm starting at the eye center and pointing in the direction of the focea by 1. Repeating these steps for all angular eye positions across recordings yielded an approximation proportional to the distribution of focea directions in spherical coordinates. Elevations in Fig. 8d were computed from the marginal distribution (i.e., after summing over all azimuth values) either as circular mean (Fig. 8d, top) or circular standard deviation (Fig. 8d, bottom) using the CircStat toolbox^61^.

To avoid that systematic changes in head tilt bias focea elevation for the different freely moving conditions, we used stratified sampling of the joint distributions of head pitch and roll values; that is, pitch and roll data for each condition were binned (5° bin size for both pitch and roll) and for each bin a random subset of samples were kept such that the frequency of pitch/roll values in that specific bin was equal across all conditions. The stratified datasets were used to compute focea elevation in Fig. 8d (20° ± 0° head-fixed, 16.3° ± 2.6° open field, 15.9° ± 2.4° social interaction, 17.0° ± 3.2° object tracking). The elevation for the control condition (“Focea fixed in head”) was computed using the same stratified data as the focea elevation (20° ± 0° head-fixed, 0.8° ± 0.8° open field, 0.8° ± 0.8° social interaction, 0.3° ± 1.3° object tracking). We repeated the same analysis with different torsion gain values (0.217 and 0.433). The precise torsion gain value had a rather small influence on focea elevation in freely moving mice: a change in torsion gain of 33% resulted in a 10% change in focea elevation (Supplemental Fig. 8d).

Optical flow fields during locomotion were computed using data from the visual object-tracking task. The task involved approaching a virtual object on a touchscreen, tracking of the object, and collection of a reward on the other side of the experiment chamber. The experiment chamber had a symmetric trapezoidal shape (width: 24 cm on the touchscreen side and 6 cm on the side with a reward spout opposite to the touchscreen; length: 18 cm; height: 20 cm). A top view camera (Waveshare RPi Camera (F) with 640 × 480 pixels at 30 Hz) centered between the trapezoid legs with a horizontal distance of 12 cm the touchscreen and 30 cm above the ground plane was used to monitor mouse behavior. The position of the animal in the environment was measured by tracking the left and right eye cameras on the animal’s head, together with the body center and the bottom corners of the environment using a deep convolutional neural network^58^. Supplementary Fig. 8c shows an example frame with tracking markers. The positions of the bottom corners were used to transform image pixels into real-world coordinates (centimeters) and to align tracked animal positions with the geometry of the environment. Positions of the tracked body parts in the ground (*x*/*y*) plane (*z* = 0 cm) were computed by correcting for the perspective of the top view camera. This required knowledge of the height of the tracked body parts. The typical height of the eye tracking cameras for a straight head (pitch = 0° and roll = 0°) was about 5 cm and we assumed that the height varied linearly with head pitch (7 cm for pitch = 90° and 3 cm for pitch = −90°). For the simulations, body height was kept fixed at 4 cm. Head orientation in the chamber was defined as the unit vector starting at the midpoint between the two eye tracking cameras and pointing to the front (i.e., orthogonal to the line connecting the two eye camera points). For a straight head, the eyes were approximately 1 cm below the midpoint between the eye tracking cameras with an interocular distance of 1 cm. Head pitch but not roll was used to find the midpoint between the eyes and left and right eye positions (relative to the midpoint between the eye cameras) and the vertical component of the head direction vector.

To compute the optical flow vectors for the left and right eyes, a grid centered around the focea for each eye was used. As a first step, periods that comprised locomotion toward the touchscreen or the reward spout were extracted from the head and body tracking data (body speed >10 cm/s and maximum absolute difference between head and body velocity <1 cm/s). Average focea azimuth and elevation for the left or right eye were computed for the extracted locomotion periods. An equally spaced grid (spacing 10°) centered at the left or right focea extending ±50° in azimuth and ±40° in elevation was created. Positions of the grid vectors in eye coordinates (i.e., in an eye reference frame) were computed as for the focea described above. For each frame, these grid vectors were transformed into vectors in absolute space using the absolute positions (*x*/*y*/*z*) of the eyes in the chamber, the orientation of the animal’s head in the chamber, and the angular eye positions along with eye torsion. The intersections of the grid vectors with the walls of the environment were computed using a virtual model of the chamber (Supplementary Fig. 8c). For each grid vector, the difference in azimuth and elevation (in the chamber reference frame) between the current and preceding frame as “viewed” from the current eye position in space (*x*/*y*/*z*) was used as a measure of local optical flow. This yielded Δazimuth and Δelevation values for each grid point for each pair of successive frames. As mouse eye movements help to stabilize the visual field (and the focea) relative to the ground, changes in azimuth and elevation in the chamber reference frame are approximately aligned with changes in azimuth and elevation in an eye-centered reference frame (up to a rotational component). For each mouse, the Δazimuth and Δelevation values for each grid point were averaged (circular mean) across all frame pairs. The flow fields in Fig. 8e show the average flow vectors across all four mice and Supplementary Fig. 8e the flow vectors for the single mice.

### Reporting summary

Further information on research design is available in the Nature Research Reporting Summary linked to this article.

## Supplementary information

Supplementary Information

Peer Review File

Reporting Summary

## Data Availability

All data are available for download and curated at the Human Brain Project Joint Platform at the following location: 10.25493/VKV1-X9C. Source Data are provided with this paper.

## Source data

Source Data

## Code availability

The custom code used to analyze the data can be found at the same location as the data given above. Example code for generating pRF maps from wide-field data is available from https://github.com/fattsmellf/Focea-pRF-Fits.
