# Supplementary material for: Mouse visual cortex contains a region of enhanced spatial resolution
Source: Nat Commun. 2021 Jun 29;12:4029. doi: 10.1038/s41467-021-24311-5 (PMC8242089; doi:10.1038/s41467-021-24311-5)
Supplement: Supplementary file 1 — Supplementary Information [file 41467_2021_24311_MOESM1_ESM.pdf]

Supplementary Information

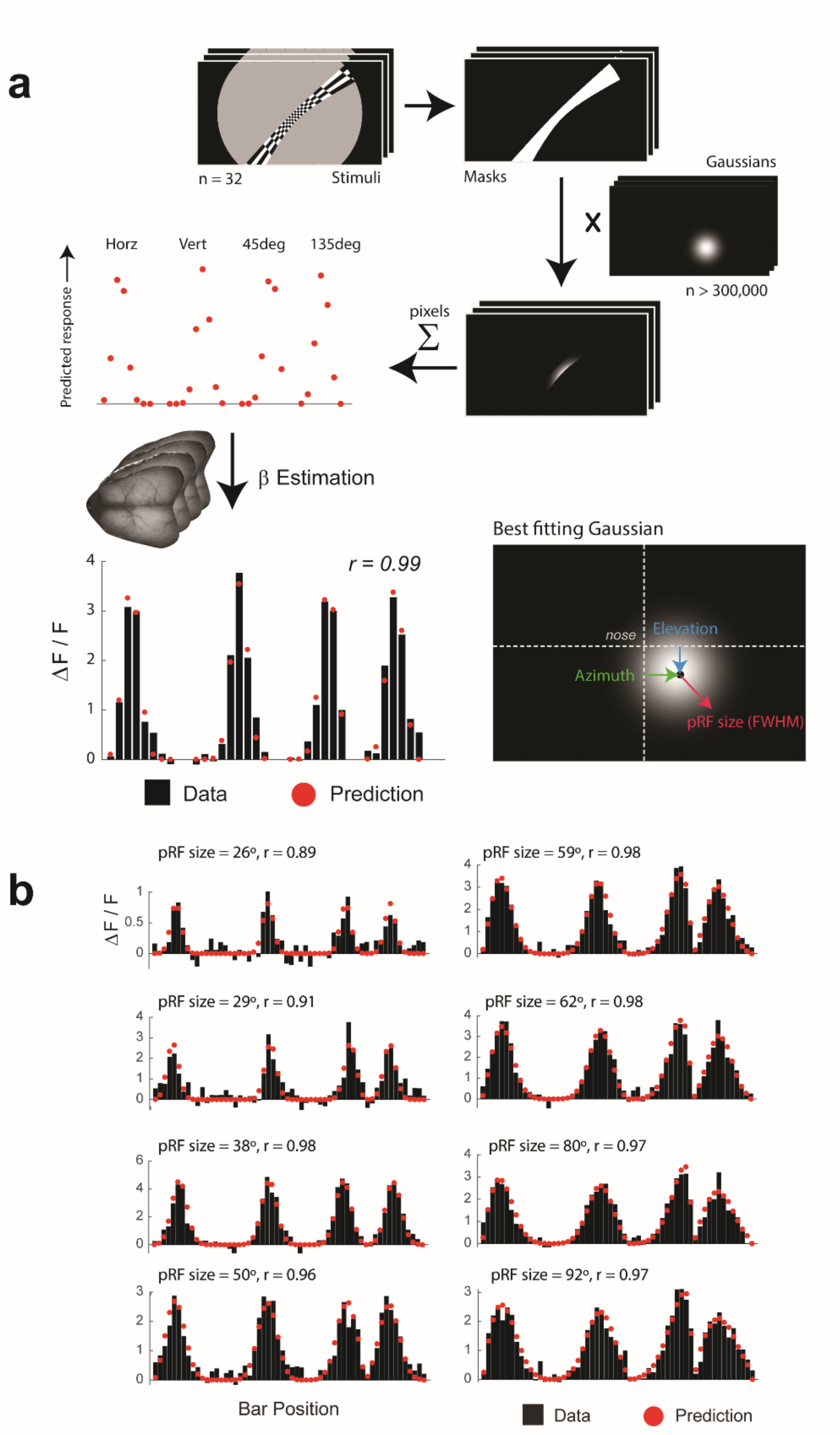

Supplementary Fig. 1. The population Receptive Field mapping (pRF) technique. **a** pRF mapping is a model-based approach to estimate the aggregate receptive field of the underlying data. Mice viewed

31 checkerboard bars of different orientations and locations (plus one blank image). These stimuli were described as masks with values of 0 (no contrast present) or 1 (contrast present). The pRF model is a forward model in which the pRF is assumed to take on a Gaussian form. More than 300,000 Gaussians were generated with different azimuths, elevations and standard deviations. Each Gaussian was multiplied by each stimulus mask to create the predicted spatial response profile, this was then summed over all pixels to create the predicted response of the Gaussian to all stimuli. These predictions were fit to the actual calcium data from different pixels in the brain image by linear regression to estimate the β weight that produced the lowest sum-of-squares error between the prediction and the response. The Gaussian that produced the lowest sum-of-squares error was taken as the pRF for that pixel. We then used the azimuth, elevation and FWHM (proportional to the standard deviation) of the best-fitting Gaussian to create the maps shown in Figure 1. **b** Example pixel responses for small (top left) to large (bottom right) pRF fits.

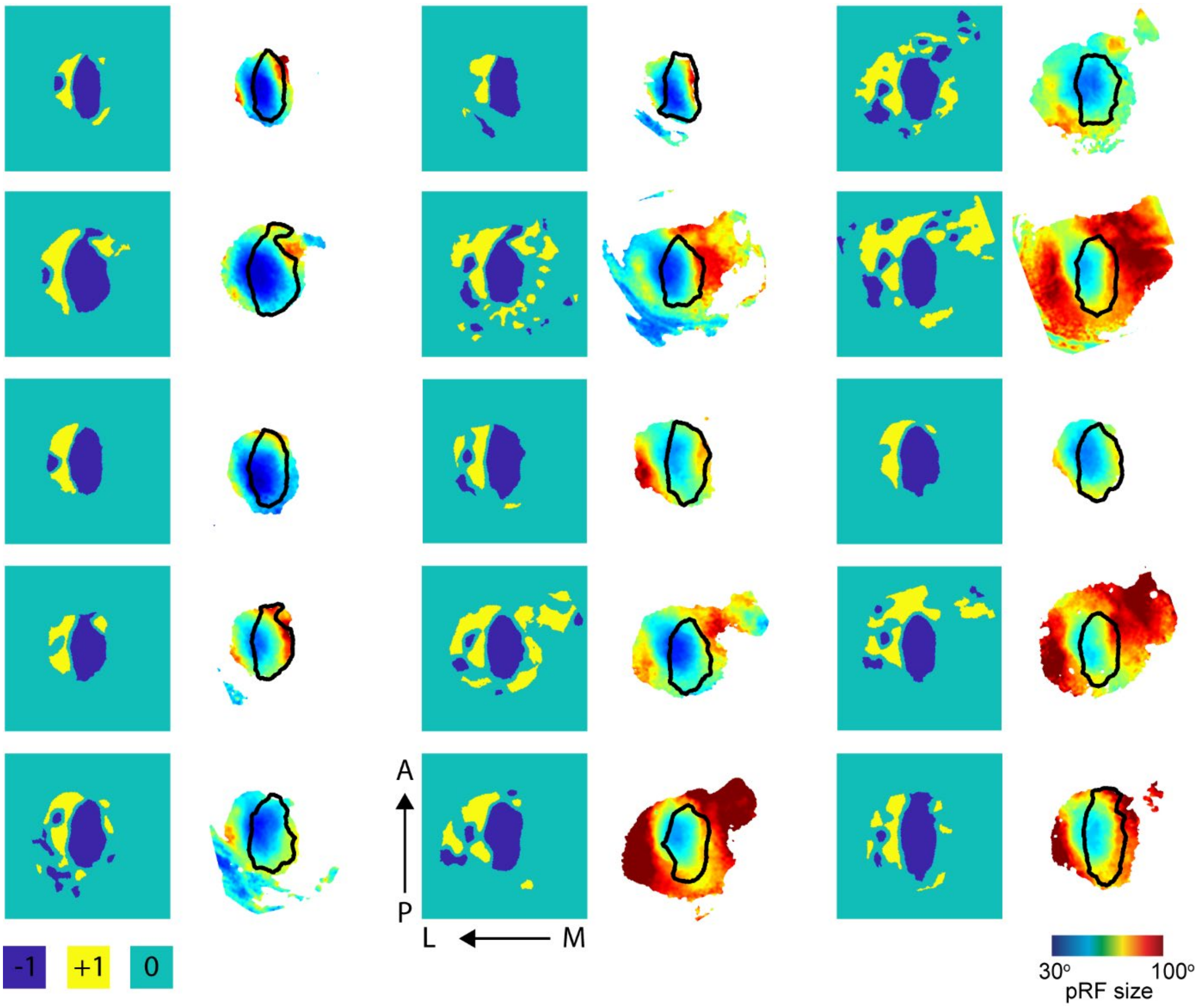

Supplementary Fig. 2. Retinotopic maps from 15 unilaterally imaged mice. For each mouse the field-sign map derived from the wide-field pRF maps is shown on the left and the map of pRF size is shown on the right. Area V1 is marked on the pRF size maps with a black outline. All maps were thresholded by the quality of the pRF model fit (at Pearson's r = 0.75).

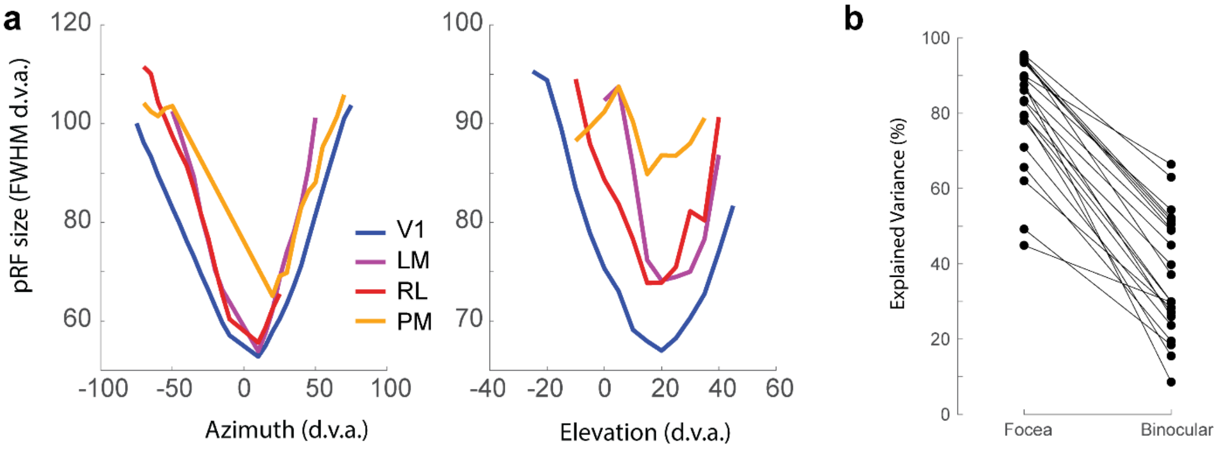

Supplementary Fig. 3. a pRFs in higher visual areas. The panels show the average pRF size binned by azimuth (left panel) and elevation (right panel) obtained from different visual areas. The visual areas were identified by field sign analysis as described in the methods section. Areas LM, RL and PM were reliably identified in the population of 11 bilaterally imaged mice. pRF size was higher in LM, RL and PM than in V1, but the relationship to the location of the pRF was qualitatively similar to that in V1. **b** Distance from the focea is better able to explain pRF sizes than binocularity. The graph shows the explained variance (r^2) of two regression models on pRF size. The first, labelled 'focea', contained a predictor with the angular distance of each pRF from the focea (labelled here as 'focea'). The second model ('Binocular') contained a categorical predictor being either 0 for monocular pRF positions or 1 for binocular pRF positions. Each dot is a hemisphere (total = 22). The focea model explained, on average, 81% of the variance of pRF size and the binocular model 37% (paired t-test, $t_{21} = 14.7$, $p << 0.001$). A full model containing both r-eccentricity and binocularity did not explain more variance than the model based on distance from the focea alone (mean explained variance = 82%, likelihood ratio test, $p>0.05$).

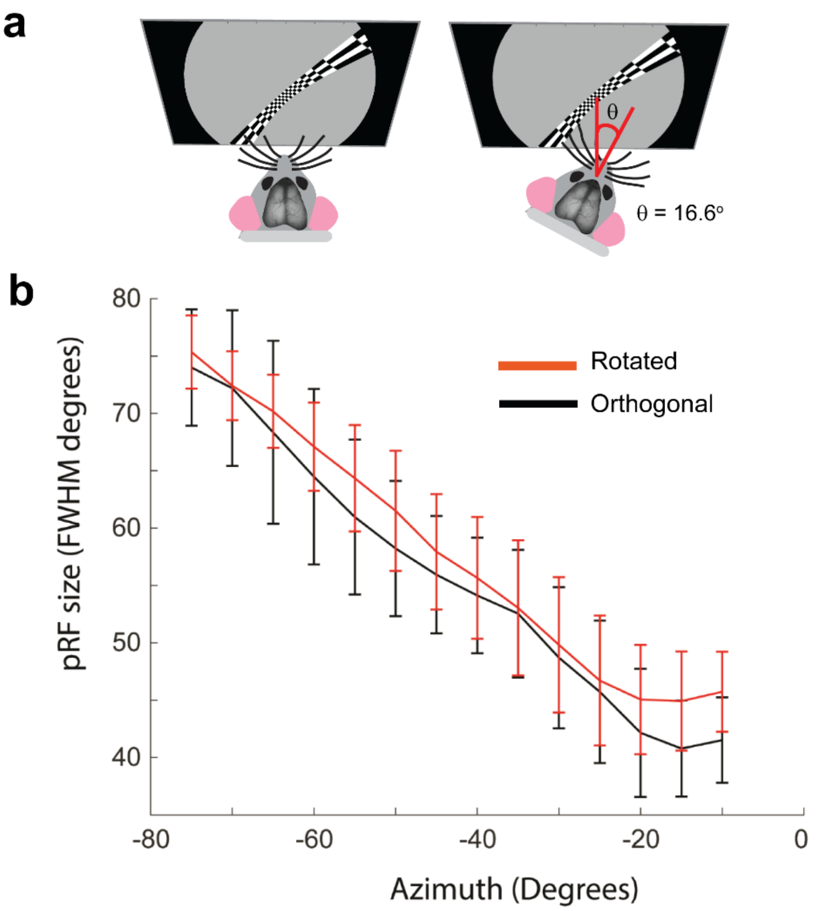

Supplementary Fig. 4. The region of small pRF size was not due to the morphing of the stimulus. **a** The visual stimuli used in this study were morphed to account for the distortion introduced by the flat LCD screen. This means the stimuli were physically smallest at azimuths/elevations located directly in front of the mouse, but covered equal areas on the retina at all azimuths/elevations. To test whether the small pRF sizes observed at the focea were an artifact of the stimulus morphing we rotated the mouse relative to the screen by 16.6^{o} and we therefore placed the smallest stimuli at an azimuth of -16.6^{o}. If the relationship between pRF size and azimuth was due to stimulus morphing, we expect a shift of the relationship between azimuth and pRF size to the left. **b** The observed data did not show this relationship and the two data sets were statistically indistinguishable (t-test on beta slope and intercept, $p > 0.05$).

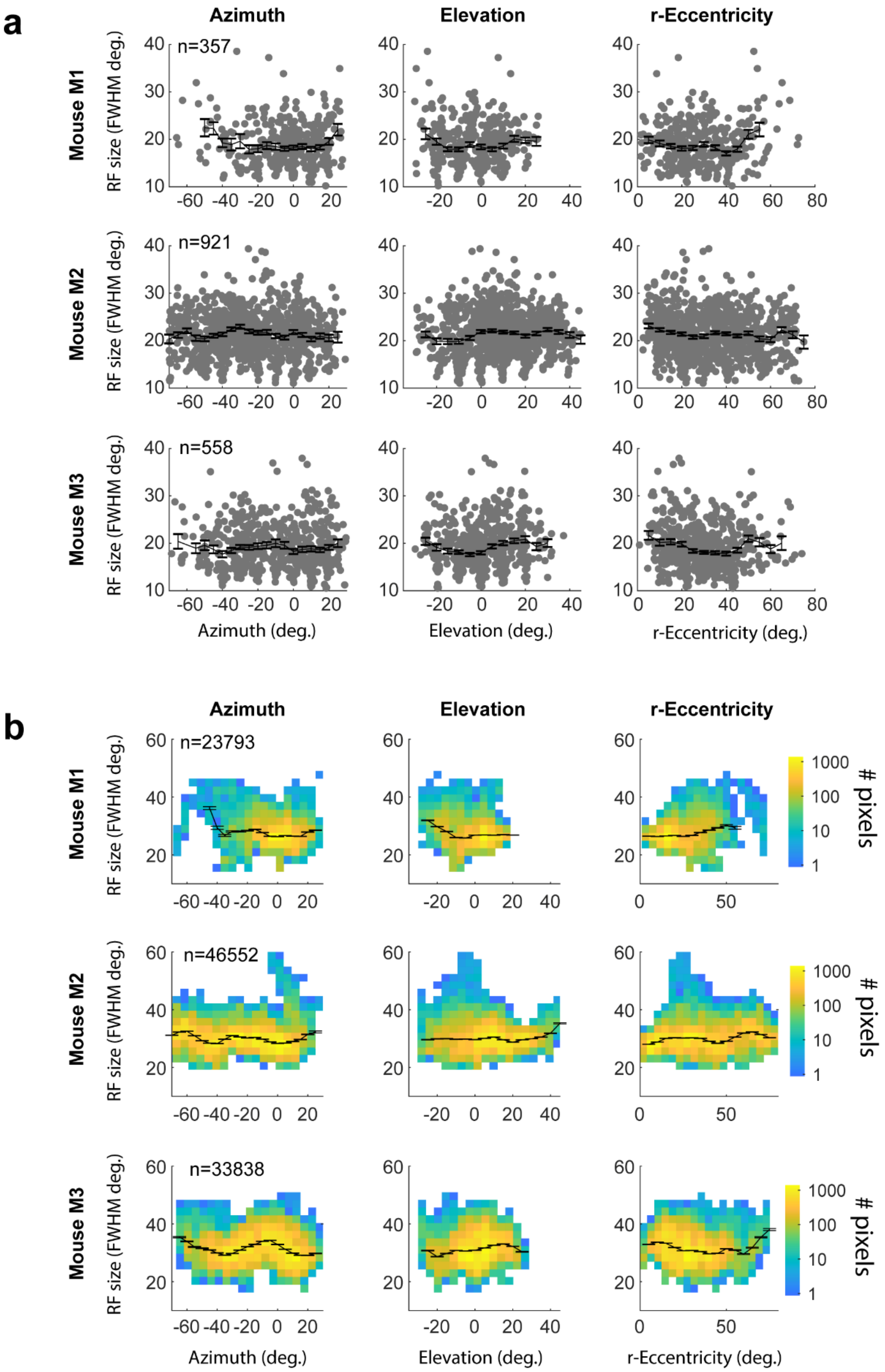

Supplementary Fig. 5. a There was no consistent relationship between the azimuth (left panel), elevation (middle panel) and r-eccentricity (right panel) of the RF of individual cells and the RF size in

the V1 two-photon imaging data in the three imaged mice. Data shown with the same format as Figure 2c. **b** No consistent relationships were found for RF size of pixels of the raw images and the azimuth, elevation or eccentricity of the raw image RF. Due to the high number of data-points they are shown here as density plots on a logarithmic scale.

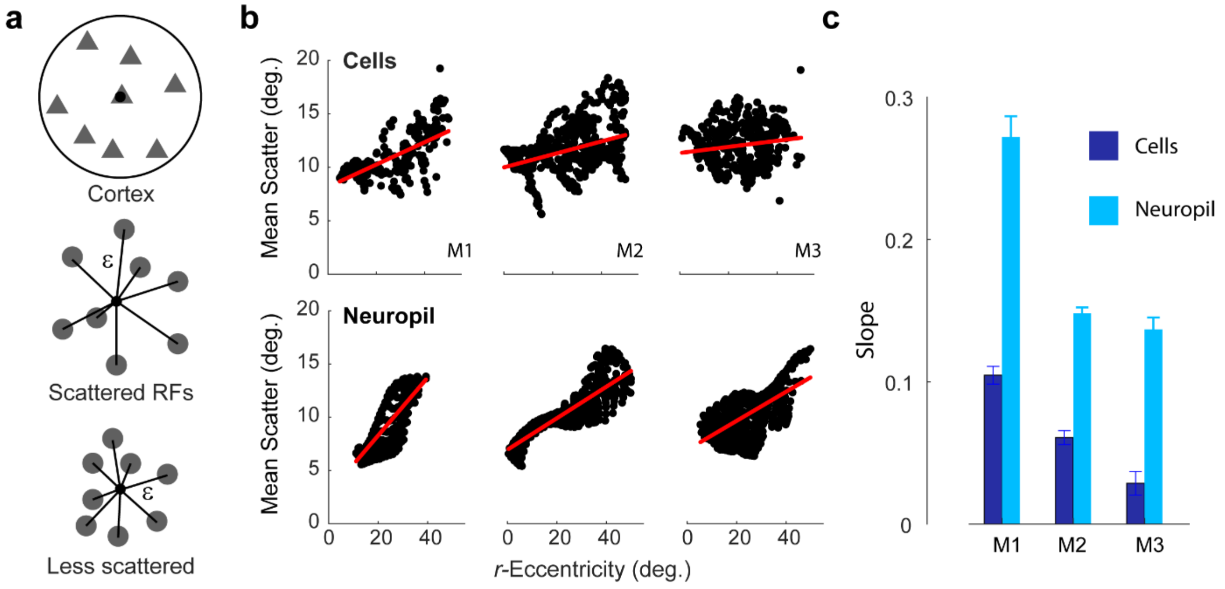

Supplementary Figure 6. The slope of the relationship between RF scatter and r-eccentricity of the neuropil is steeper than that of single cells. **a** To quantify scatter we drew analysis windows of 400um radius on the cortical surface for both the single-cell and neuropil datasets (upper panel). We measured the Euclidean distance of each cell/pixel's RF from the aggregate RF of all cells/pixels in the window (ε) and calculated the mean value. This value will be larger for more scattered representations in the periphery (middle panel) than the clustered representations we expect to see at the focea (lower panel). **b** (upper panel) The relationship between the r-Eccentricity of the aggregate RF (i.e. the spherical angle from the focea) and the mean value of ε as described above for single-cell data from the three mice in which we measured most of V1 using two-photon imaging. The red line shows the best-fitting regression slope. (lower panels) The same as above, but for the neuropil data. The neuropil data was inherently less scattered than the single-cell data and the regression slopes were steeper. **c** Summary of the slopes for the three mice, error bars indicate 1 s.e.m.

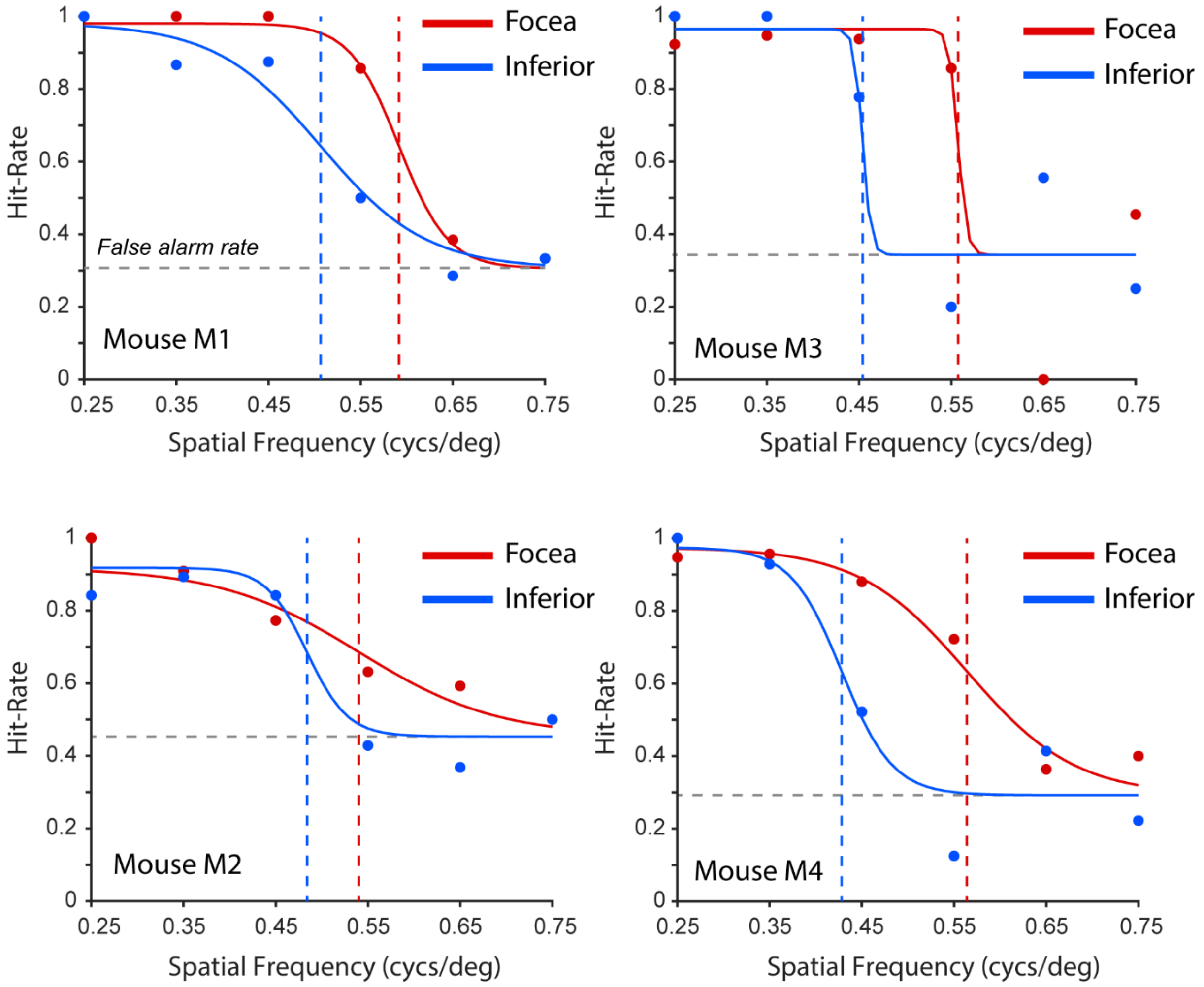

Supplementary Fig. 7. Spatial frequency detection curves for all four mice. The data-points are the average hit-rates across sessions for each tested spatial frequency. Acuity at the focea was higher (spatial frequency threshold is indicated by the red dashed line) than in the inferior visual field (dashed blue line). The curves are fits of logistic functions as described in the main text.

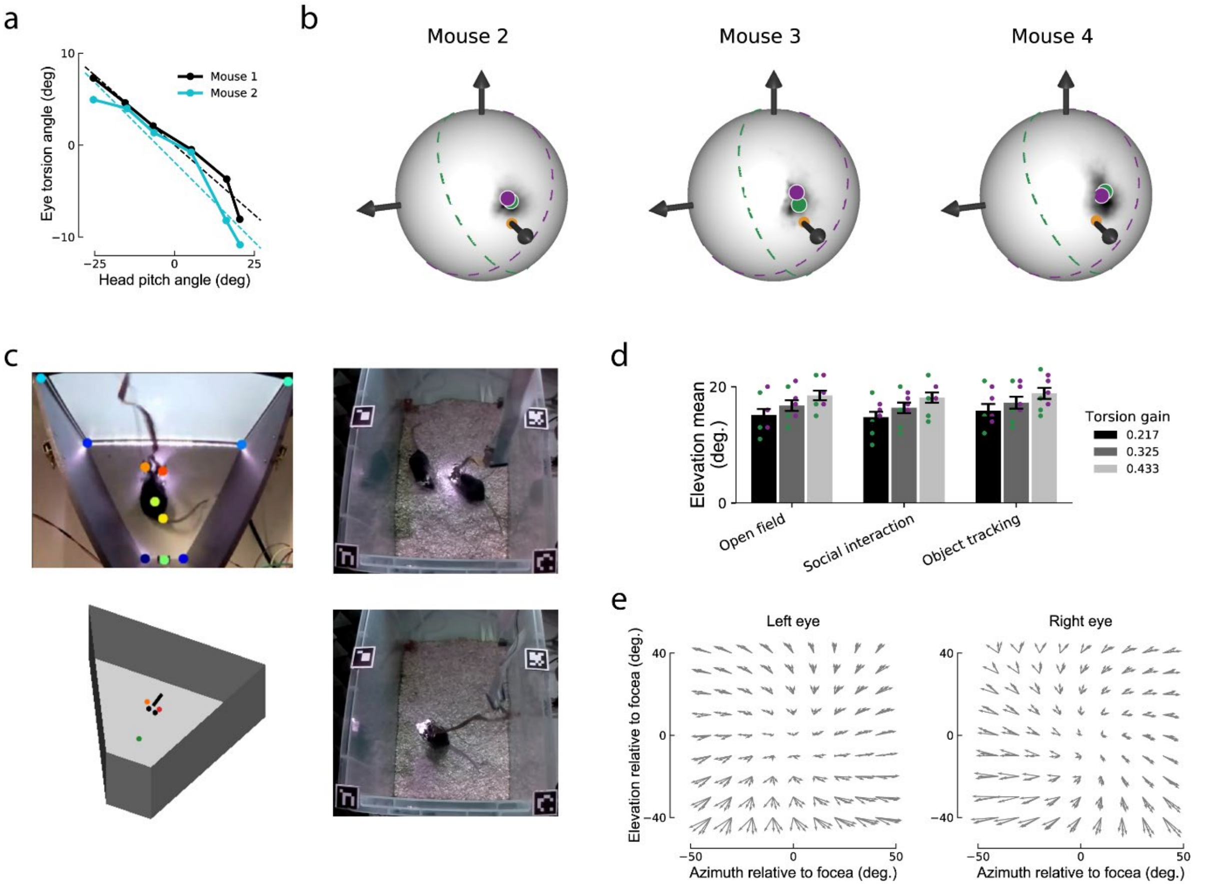

Supplementary Fig. 8. Eye tracking in freely moving mice. **a** Relation between head pitch and eye torsion for the right eye of two passively tilted mice. The relation is approximately linear (Pearson correlation of raw data; mouse 1, r=-0.48; mouse 2, r=-0.53) and the negative slope indicates counter-rotation of the eye relative to the head. **b** Distributions of the focea in three freely moving mice, format as in Figure 8b of the main text. **c** Top left: example video frame from the top view camera during the visual object tracking task. Colored dots indicate positions of tracked parts including left and right eye cameras on the animal’s head (orange and red), body center (light green) and the bottom corners of the environment (blue). Bottom left: top and diagonal views of the tracked parts used for optical flow computation placed within a model of the touchscreen chamber. The black line indicates 3D head orientation and the black dots the positions of the left and right eyes in the chamber. Top right: example frame from the top-view camera during the social interaction task. Bottom right: example frame from the open field task. **d** Recomputation of the elevation of the focea using a range of torsion values from 0.217 to 0.433. Changing the torsion value had little effect

on our measure of the elevation of the focea during the three tasks (format as in Figure 8d of the main text). **e** Optical flow fields during locomotion for the left and right eyes. Gray arrows show optical flow directions for the single mice. Data from 4 mice.
