# Supplementary material for: Mouse visual cortex contains a region of enhanced spatial resolution
Source: Nat Commun. 2021 Jun 29;12:4029. doi: 10.1038/s41467-021-24311-5 (PMC8242089; doi:10.1038/s41467-021-24311-5)
Supplement: Supplementary file 3 — Reporting Summary [file 41467_2021_24311_MOESM3_ESM.pdf]

nature research

Corresponding author(s): Pieter Roelfsema
Matthew Self

Last updated by author(s): Jan 8, 2021

Reporting Summary

Nature Research wishes to improve the reproducibility of the work that we publish. This form provides structure for consistency and transparency in reporting. For further information on Nature Research policies, see our Editorial Policies and the Editorial Policy Checklist.

Statistics

For all statistical analyses, confirm that the following items are present in the figure legend, table legend, main text, or Methods section.

n/a	Confirmed	
☐	☒	The exact sample size (*n*) for each experimental group/condition, given as a discrete number and unit of measurement
☐	☒	A statement on whether measurements were taken from distinct samples or whether the same sample was measured repeatedly
☐	☒	The statistical test(s) used AND whether they are one- or two-sided<br>*Only common tests should be described solely by name; describe more complex techniques in the Methods section.*
☐	☒	A description of all covariates tested
☐	☒	A description of any assumptions or corrections, such as tests of normality and adjustment for multiple comparisons
☐	☒	A full description of the statistical parameters including central tendency (e.g. means) or other basic estimates (e.g. regression coefficient) AND variation (e.g. standard deviation) or associated estimates of uncertainty (e.g. confidence intervals)
☐	☒	For null hypothesis testing, the test statistic (e.g. F, t, r) with confidence intervals, effect sizes, degrees of freedom and P value noted<br>*Give P values as exact values whenever suitable.*
☒	☐	For Bayesian analysis, information on the choice of priors and Markov chain Monte Carlo settings
☒	☐	For hierarchical and complex designs, identification of the appropriate level for tests and full reporting of outcomes
☐	☒	Estimates of effect sizes (e.g. Cohen's *d*, Pearson's *r*), indicating how they were calculated

Our web collection on statistics for biologists contains articles on many of the points above.

Software and code

Policy information about availability of computer code

Data collection	Encephalos v1.0 (Caenotec-Prof. Ralf Schnabel): Wide field imaging microscope control and image capture. Custom code available from Prof. Ralf Schnabel: r.schnabel@tu-bs.de<br><br>COGENT graphics v1.33 (developed by John Romaya at the LON at the Wellcome Dept. of Imaging Neuroscience): graphics control for all experiments. Freely avialable from http://www.vislab.ucl.ac.uk/cogent_graphics.php<br><br>MATLAB 2019b (Mathworks Inc.): Experiment control scripts. Commercial.<br><br>OpenEx v2.31 (TDT technologies): Data acquisition software for Electrophysiological data. Commercial.<br><br>Scanbox v2 (Neurolabware): Two-photon microscope control and image acquisition. Commercial.
Data analysis	MATLAB 2019b (Mathworks Inc.): Experiment control scripts. Commercial.<br><br>CAIMAN v1.8.8 (Flatiron Institute): Two-photon image processing. Available from https://github.com/flatironinstitute/CaImAn-MATLAB/wiki/Component-classification-with-a-convolutional-neural-network

pRF analysis: Custom Code, available from https://github.com/fattsmellf/Focea-pRF-Fits

Electrophysiological analysis: Custom code available from HBP (see below).

Two-photon analysis: Custom code available from HBP (see below).

For manuscripts utilizing custom algorithms or software that are central to the research but not yet described in published literature, software must be made available to editors and reviewers. We strongly encourage code deposition in a community repository (e.g. GitHub). See the Nature Research guidelines for submitting code & software for further information.

Data

Policy information about availability of data

All manuscripts must include a data availability statement. This statement should provide the following information, where applicable:
- Accession codes, unique identifiers, or web links for publicly available datasets
- A list of figures that have associated raw data
- A description of any restrictions on data availability

All data and the computer code used to analyze the data are available for download and curated at the Human Brain Project Joint Platform at the following DOI: 10.25493/VKV1-X9C. Example code for generating pRF maps from wide-field data is available at the same location and from https://github.com/fattsmellf/Focea-pRF-Fits. Source data are provided with this paper.

Field-specific reporting

Please select the one below that is the best fit for your research. If you are not sure, read the appropriate sections before making your selection.

☒ Life sciences ☐ Behavioural & social sciences ☐ Ecological, evolutionary & environmental sciences

For a reference copy of the document with all sections, see nature.com/documents/nr-reporting-summary-flat.pdf

Life sciences study design

All studies must disclose on these points even when the disclosure is negative.

Sample size	No sample size calculations were made. The wide-field images were collected for a separate study, post-hoc power analysis indicated that we had a power of > 0.9.The electrophysiological dataset was similarly collected for different studies, the dataset was very large and power was greater than 0.99. For two-photon and behavioral data we report statistics in individual animals.
Data exclusions	No mice were excluded from the wide-field data. Individual recording contacts with poorly fitting receptive fields were excluded as described in the manuscript. Individual cells with poorly fitting receptive fields were excluded from the two-photon data as described in the analysis.
Replication	No replication attempts were made. All receptive-field measurements were assessed using a bootstrapped reliability index to ensure that the receptive-fields could not have arisen due to chance activations or noise. This combined with the high power ensures a very high reliability. Our ethical protocols do not allow us to run experiments purely for replication purposes.
Randomization	No between groups comparisons were made so randomization was not possible.
Blinding	Experimenters were not blinded as each experiment consisted of only a single group. It is therefore not possible to blind the experimeneter to group allocation.

Reporting for specific materials, systems and methods

We require information from authors about some types of materials, experimental systems and methods used in many studies. Here, indicate whether each material, system or method listed is relevant to your study. If you are not sure if a list item applies to your research, read the appropriate section before selecting a response.

Materials & experimental systems

n/a	Involved in the study	
☒	☐	Antibodies
☒	☐	Eukaryotic cell lines
☒	☐	Palaeontology and archaeology
☐	☒	Animals and other organisms
☒	☐	Human research participants
☒	☐	Clinical data
☒	☐	Dual use research of concern

Methods

n/a	Involved in the study	
☒	☐	ChIP-seq
☒	☐	Flow cytometry
☒	☐	MRI-based neuroimaging

Animals and other organisms

Policy information about studies involving animals; ARRIVE guidelines recommended for reporting animal research

Laboratory animals	Mus Musculus. C57Bl/6J, Thy1-GCamP6f. Both sexes used. Age range: 2-14 months.
Wild animals	No wild animals were used.
Field-collected samples	No field-collected samples were used.
Ethics oversight	Animal Ethical Committee of the Royal Netherlands Academy of Arts and Sciences (Netherlands)<br>Centrale Commissie Dierproven (Netherlands)<br>UK Home Office (UK)

Note that full information on the approval of the study protocol must also be provided in the manuscript.
