# Supplementary material for: Mouse visual cortex contains a region of enhanced spatial resolution
Source: Nat Commun. 2021 Jun 29;12:4029. doi: 10.1038/s41467-021-24311-5 (PMC8242089; doi:10.1038/s41467-021-24311-5)
Supplement: Supplementary file 2 — Peer Review File [file 41467_2021_24311_MOESM2_ESM.pdf]

Reviewer #1 (Remarks to the Author):

This is a very interesting study documenting a non-uniform spatial organization of receptive fields in mouse V1. The authors used wide-field imaging and modeling to infer that population receptive fields (pRF) sizes as a function of azimuth and elevation. They find that there is a location central location (0 deg azimuth and 20 deg elevation), which the authors term the 'focea', where sizes are minimum, and they increase with eccentricity measured form this point.

Two-photon imaging revealed the changes in pRF are a consequence of changing RF scatter and an over representation of the binocular region. The authors further show that behavioral performance in a go/no-go task is better in the focea, and make eye movements to keep this region near the focus of expansion of the optic field during locomotion. Overall, these data contribute important new aspects of the spatial organization of the mouse visual system that will be of wide interest to the community.

I only have some minor comments/questions:

1) The analysis of two-photon data at the single cell level, or by simply smoothing the raw images, yielded different slopes for the relationship between pRF and r-eccentricity. My intuition, contrary to that of the authors, was that the scatter for the single cells data would be higher than that of smoothed, raw images. After all, the maps for elevation and azimuth for the raw images look rather smooth (Fig 5). The opposite was true, which the authors expected simply because such analysis would mimic the wide-field imaging data. A more detailed explanation (or better yet, a specific model) for why the inclusion of the neuropil yields higher RF scatter would make the paper stronger.

2) The behavioral data appears to have been collected with both eyes open. In this case, one would expect performance within the binocular field of view to be better than in monocular areas simply due to binocular summation. Have the authors repeated the go/no-go experiments under monocular conditions? If so, are the results the same?

3) Is it possible that the decreased scatter within the binocular zone is simply due to binocular matching together with a larger cortical magnification? What would happen, for example, if the authors were to repeat the experiments on mice reared with mis-aligned eyes (presumably yielding only monocular neurons)? Will the scatter difference disappear ?

Altogether, the non-uniformity of scatter of RFs in V1, along with its consequences for the organization of downstream areas and behavior are an important contribution to the field.

Reviewer #2 (Remarks to the Author):

This manuscript elegantly synthesizes multiple methods, including widefield imaging, electrophysiology, 2-photon calcium imaging, and computational modeling, to provide a wholistic description of the relationship between mouse retinotopic organization, receptive field size and scatter across single cell and population measures. Importantly, this combination of techniques demonstrates a novel finding of a region of increased spatial resolution in the visual field directly in

front of and above the mouse, the focea. The authors find a reduction in population receptive field (pRF) size at the focea, due to an increasingly ordered representation of visual space with reduced scatter in single cell RF positions in this region, beyond an increase in cortical magnification, rather than a reduction in single cell RF size as one might naively expect.

The results described in Figures 1-5 demonstrate the existence of the focea with widefield imaging, then go on to show with electrophysiology and single cell 2-photon calcium imaging that the reduction in population receptive field size at the focea is not due to a reduction in single cell receptive field size, but rather is associated with reduced scatter in RF position as well as an increase in the amount of cortical territory dedicated to this part of space. The results are quite convincing, especially given the large size of the widefield and electrophysiological datasets. In addition, these results provide important insight into the nature of the widefield signal and pRF measures generally, demonstrating that population RF sizes measured with this method arise from scatter in both cellular and neuropil signals over a 200-400um region, whereas signals from cell bodies (from single cell 2-photon or MUA with ephys) only cannot fully account for the observed pRF sizes in widefield data. This is a valuable insight for the field and should be considered in the interpretation of widefield results and in any assumptions of how widefield signals translate to single cell properties.

In contrast to the lack of systematic changes in single cell RF size across V1, in figure 6 the authors demonstrate a relationship between single receptive field size and eccentricity from the focea in several higher visual areas. The authors suggest that the reduction in population RF size in V1 and associated reduction in RF scatter could translate to smaller RFs in higher visual areas, contributing to higher spatial resolution vision in the focea. The mechanisms of such a transformation remain unexplored.

The authors move beyond characterization and delve into the functional implications of the measured relationships during freely moving and trained behaviors. Using a visual detection task, the authors demonstrate increased spatial acuity in the foceal region of space, marked by higher spatial frequency thresholds (and thus increased resolution) for stimuli directly in front of and slightly above the mouse. These results confirm the behavioral relevance of their physiological results and are a key component of the paper. Using a newly developed system for tracking head and eye movements in freely moving mice, they show that mice use compensatory eye movements to stabilize the focea on the region of space directly in front of and slightly above the mouse. This was true in multiple diverse behavioral contexts including exploration of an open field, social interaction, and object tracking on a screen. The authors suggest that the utility of keeping the focea stable in this location is to facilitate accurate object identification and navigation in the context of optic flow during locomotion.

Overall, the experiments are rigorously performed, well grounded in the literature, include appropriate controls, and are clearly described with compelling visualizations and analysis. The combination of functional and behavioral measures, along with modeling and thorough computational analysis, provides a comprehensive view of the structure and function of a focea for high acuity vision in the mouse, including evidence for the behavioral utility of the focea. These results clearly provide a valuable addition to our understanding of mouse visual cortex. I recommend that the manuscript be accepted with minor revisions, per the specific comments below.

Specific comments

General

The authors may wish to reference these relevant studies and discuss how their results relate to previous findings:

Michaiel, A. M., Abe, E. T. T. & Niell, C. M. Dynamics of gaze control during prey capture in freely moving mice. Elife 9, 1–27 (2020). https://elifesciences.org/articles/57458

Oommen, B. S. & Stahl, J. S. Eye orientation during static tilts and its relationship to spontaneous head pitch in the laboratory mouse. Brain Res. 1193, 57–66 (2008). https://pubmed.ncbi.nlm.nih.gov/18178173/

Waters, J. et al. Biological variation in the sizes, shapes and locations of visual cortical areas in the mouse. PLoS One 14, 1–13 (2019). https://journals.plos.org/plosone/article?id=10.1371/journal.pone.0213924

Zhuang, J. et al. An extended retinotopic map of mouse cortex. Elife 6, (2017). https://elifesciences.org/articles/18372

Regarding this last publication, one interesting point of discussion is the implication of the reduced scatter and pRF size at the focea and the organization of areal boundaries in this zone. As demonstrated in Figure 1 as well as in Zhuang et al. (Figures 4&5), the boundary between V1, LM, AL, and RL does not always come to a clear meeting point, and the lateral V1 boundary measured with functional imaging does not match the anatomical boundary of V1 measured with cytoarchitecture or histological markers. How might the size and scatter of RF locations in the binocular zone of V1 and neighboring areas relate to the ability to accurately map areal boundaries with widefield imaging, and to anatomical boundaries based on cellular architecture and connectivity?

Main text and figures

- The text is very well written and clear, particularly in the description of results for Figures 1-5. Results and discussion for Figures 6-8 regarding functional implications of the foceal representation could be elaborated to provide additional context and motivation for these experiments, how they relate to the findings in Figures 1-5, and their practical significance. The inclusion of behavior is very valuable to the study and it's impact should be emphasized.
- Throughout the text, ensure that references to population RFs vs. single cell RFs are abundantly clear, as they can easily be confused and the interpretation is very different depending on which is being discussed.
- Keep in mind that the various spatial reference frames and transformations between them can be difficult to keep track of for those not familiar with these types of experiments, especially for eye tracking in freely moving animals. Clarify any relevant text as necessary.
- Line 64, and generally – why did the authors choose to distinguish the focea from the fovea of primates? Why use the term 'focea'? Some context would be helpful to the reader here. Are there other species with a focea? Is this a novel term or is there precedence for its use?

• Line 67 – please elaborate on description of r-Eccentricity and how it is calculated based on altitude and elevation.
• Figure 1 – it would be valuable to provide the retinotopic maps for all mice recorded as a supplemental figure, so that the reader can gain an intuition for the variability in the size, shape, and layout of the areas (ex: Waters et al., 2019).
• Figure 4d – please describe r-Eccentricity in the figure legend as this is the first time it is mentioned in this figure.
• Line 111 – states that mice that were imaged were injected with AAV-GCaMP6f, however the first section of the methods states that Thy1-GCaMP6f mice were used for 2-photon experiments
• Line 136 – typo, I believe that "included" shoud be "including" here
• Figure 5a, I believe the title for the far right panel should read "RF size" rather than "pRF size" as the measure is per pixel of the image rather than an aggregate over a window.
• Figures 4 and 5 – it would be good to show a quantification of RF size as a function of azimuth, elevation and r-Eccentricity for the 2-photon data, similar to what is shown in figure 2C for the electrophysiology data, to confirm that RF sizes are not changing drastically with distance from the focea. For instance in Figure 5a, right panel, there does appear to be some variation in RF size in portions of the image. It would be helpful to know whether there is any systematic relationship to retinotopic position, and whether any effect is consistent across mice. This could be a supplemental figure.
• Figure 5 – intermediate plots for the scatter analysis, such as the residuals as a function of eccentricity, akin to what is showin in Figure 4d, could be provided as supplemental material to support the summarized results for the pixel-wise analysis in Figure 5b&c.
• Line 160-161 – please elaborate on why one might predict that decreased scatter in the focea would result in smaller RFs in downstream areas and why this is of functional significance, to provide more context to the reader.
• Line 170 – specify whether "reduced RF size" refers to single cell RF size or population RF size, as these have different implications for the question at hand.
• The implications of the results shown in Figure 6 should be more thoroughly described in the results and in the discussion. The idea that reduced scatter in V1 is converted to smaller RFs and higher spatial resolution in higher visual areas is intriguing, but not conclusively demonstrated by the results presented. As such, the conditions that would need to be present to give rise to this situation (i.e. the sampling strategy of neurons in higher visual areas) should be discussed to provide context to the reader, and to provide suggestions for future experiments to confirm these predictions.
• In addition, the result that LM, AL and AM show this relationship between RF size and eccentricity, but RL and PM do not, should be further discussed. Why might some areas require better acuity at the focea and others do not? What does this suggest for the functional role of those areas?
• Figure 7b – it wold be good to show the full spatial frequency detection curves for all mice in the study, in addition to or rather than a single example. If these results are too noisy, adding additional mice is suggested.
• Results are only shown for one mouse in Figure 8. Results from all mice should be reported, especially for the results in Figure 8b.
• Please add a legend for bar colors to Supplemental Figure 4B for clarity.
• One of the more unique and valuable aspects of this work is the measurement of eye position across multiple behavioral contexts as it relates to the focea, however these results are buried in the supplemental matrial and are not as thoroughly explored as they could be. Supplemental Figure 4B should be moved to the main figures (Figure 8). Additional analysis of eye position in these various context would also be valuable. For example, how do head and eye position change as the mouse approaches another mouse? The dynamics of these interactions are interesting and the data is

there.
• Images depicting the experimental conditions for open field and social interaction should be provided in addition to the representation of object tracking in Supplemental Figure 4C, to provide context for Supplemental Figure 4B. Again, all relevant figure panels to this analysis would be better positioned in the main figures.

Methods:

• Overall, very thorough description of methods used, all very rigorous, well controlled, and consistent with previous literature.
• Throuought the methods, it would be helpful for novice readers if the equations were described in words, in addition to the formula being provided.
• Line 647 – which painkillers were provided?
• Line 651 – I believe the authors meant to say "as described below" rather than "above" in reference to methods for pRF mapping
• Lines 651-654, the authors describe injecting animals with AAV prior to imaging higher visual areas with 2-photon calcium imaging, however in the "Animals" section of the Methods, they describe using Thy1-GCaMP6f mice for the 2-photon imaging experiments. Please clarify which methods were used.
• Line 665 – "Hz" is repeated twice. Also the authors state that pupil was tracked at "50/100Hz", do they mean either 50 or 100Hz?
• If the authors tracked the pupil during wide field imaging with the head level, did they observe that the center of gaze was 20 degrees above the horizon (as in Oommen & Stahl, 2008)? Can they report their eye position measurements for these experiments?
• Line 667 is a bit confusing– "to ensure that check-size in degrees of visual angle was constant", perhaps it could be rephrased as "to ensure that the size of the check in degrees…"
• Why not use an open source spike detection algorithm, such as Kilosort, instead of thresholding?
• Line 943 – specify DeepLabCut in the text in addition to citing the reference
• Line 907 - how is the focea defined? This is described in the results but would be helpful to summarize here.
• I do wonder why the authors did not do an analysis of eye torsion with their pupil tracking data in the freely moving animals, similar to what was done in ref. 52. Perhaps there was some barrier to doing this analysis properly? If not, it could be suggested that they include that analysis and use directly measured torsion values. It seems they should have the necessary data to do this analysis.
• In addition, I can think of a few ways that the authors could provide increased confidence in their torsion measurements and the overall approach. First, they could perform the measurement of torsion in multiple head fixed mice, rather than just one, to give a sense of the variability across mice in Supplemental Figure 4a and confirm that the relationship is always linear and that the slope is similar across mice. Second, they could re-evaluate their model with varying values of the slope of the relationship of eye torsion to head pitch, such as 0.2 or 0.4, in addition to their used value of 0.3, to assess how sensitive their results are to this value. If slight variation in the slope used in the model does not significantly impact their results and interpretation, it would give confidence that their approach is not a major confound.
• I noticed that they may have used the wrong reference on line 959 of the methods – here they refer to ref. 53 regarding torsion measurements in rats, but I believe those measurements were actually done in ref 52.

REVIEWER COMMENTS

Reviewer #1 (Remarks to the Author):

This is a very interesting study documenting a non-uniform spatial organization of receptive fields in mouse V1. The authors used wide-field imaging and modeling to infer that population receptive fields (pRF) sizes as a function of azimuth and elevation. They find that there is a location central location (0 deg azimuth and 20 deg elevation), which the authors term the 'focea', where sizes are minimum, and they increase with eccentricity measured form this point.

Two-photon imaging revealed the changes in pRF are a consequence of changing RF scatter and an over representation of the binocular region. The authors further show that behavioral performance in a go/no-go task is better in the focea, and make eye movements to keep this region near the focus of expansion of the optic field during locomotion. Overall, these data contribute important new aspects of the spatial organization of the mouse visual system that will be of wide interest to the community.

We would like to thank the reviewer for their careful reading of the manuscript and their positive comments.

I only have some minor comments/questions:

1) The analysis of two-photon data at the single cell level, or by simply smoothing the raw images, yielded different slopes for the relationship between pRF and r-eccentricity. My intuition, contrary to that of the authors, was that the scatter for the single cells data would be higher than that of smoothed, raw images. After all, the maps for elevation and azimuth for the raw images look rather smooth (Fig 5). The opposite was true, which the authors expected simply because such analysis would mimic the wide-field imaging data. A more detailed explanation (or better yet, a specific model) for why the inclusion of the neuropil yields higher RF scatter would make the paper stronger.

This is an interesting point. We indeed reasoned that by smoothing the raw two-photon images and applying analysis windows of different sizes we could effectively simulate a wide-field signal and therefore we expected the slope of the relationship between pRF size and r-eccentricity to be closer to the wide-field values. The reviewer's question makes us realise that this assumption should be formalised.

We examined the reason for the steeper slopes for the neuropil data by comparing the level of scatter between the single-cell and neuropil datasets. For both datasets we drew analysis windows on the cortical surface and calculated the centre of the aggregate RF of the cells/neuropil that fell within the window. We took the mean of the Euclidean distances from each cell/pixel's RF to the aggregate RF. This provides a direct measure of the scatter of the RFs within the analysis window. The results are shown in Reviewer Figure R1 and have been added to the manuscript as part of Supplementary Figure 6. As anticipated by the reviewer, the neuropil maps were smoother than the single-cell maps and scatter was lower. Yet, the neuropil scatter depended more strongly on r-eccentricity. Accordingly, the slopes for the neuropil were consistently more than double the slopes for the cell bodies across the three mice. As pRF size is directly related to the level of scatter (Figure 3) the increased slope of scattering accounts for increased slope of the relationship between pRF

size and r-eccentricity for the neuropil data. We have added a description of these results to the paper on lines 167-169 and as Supplementary Figure 6.

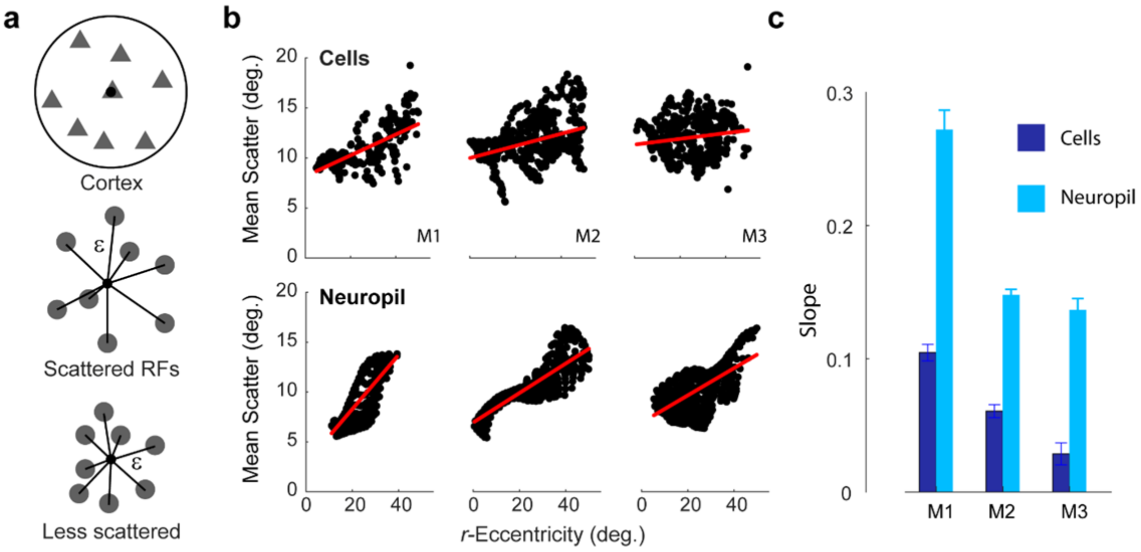

Figure R1. The neuropil shows a steeper relationship between RF scatter and r-eccentricity than single cells. a (**upper**) To quantify scatter we drew analysis windows of 400um radius on the cortical surface for both the single-cell and neuropil datasets. We took the Euclidean distance of each cell/pixel's RF from the aggregate RF of all cells/pixels in the window (ε) and calculated the mean value. This value will be larger for more scattered representations in the periphery (**middle**) than the clustered representations we expect to see at the focea (**lower**). **b** (**upper**) The relationship between the r-Eccentricity of the aggregate RF (i.e. the spherical angle from the focea) and the mean value of ε as described above for all three mice in which we tiled V1 using two-photon imaging. The red line shows the best-fitting regression slope. (**lower**) The analysis for the neuropil data. The neuropil data is inherently less scattered than the single-cell data, but the regression slopes were steeper. **c** Summary of the slopes for the three mice, errorbars indicate 1 s.e.m.

2) The behavioral data appears to have been collected with both eyes open. In this case, one would expect performance within the binocular field of view to be better than in monocular areas simply due to binocular summation. Have the authors repeated the go/no-go experiments under monocular conditions? If so, are the results the same?

The reviewer is correct that it is difficult to compare behaviour at lateral (monocular) locations with that at the central (binocular) locations, both because of probability summation for binocular compared to monocular detectors, and also because the viewing angle for lateral locations is higher than for central locations which reduces the luminance of stimuli presented at lateral locations. For both of these reasons we have focussed on the comparison of spatial frequency thresholds at the two central locations which both fell within the binocular zone (Figure R2) and have similar viewing angles. The difference in behavioural thresholds between the central stimulus in the upper field and the lower field demonstrates the benefits of presenting stimuli in the focea. We now explain this issue more clearly in the results section on lines 199-204 and note that the results at lateral locations should be interpreted with caution. We note that re-running the experiment with monocular

viewing would not solve the issues with the viewing angle of the LCD screen.

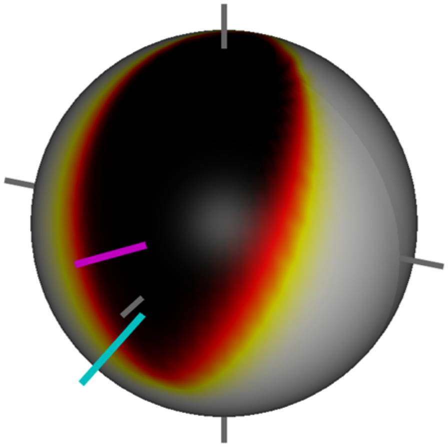

Figure R2. Eye-movement recordings in head-fixed mice show that the central positions tested in the behavioural experiment fall within the binocular zone. The color scale shows the probability that a region of the visual scene fell was viewed binocularly (darker colors = higher probability). The focea at [azi = 0, ele = 20] fell within the region of binocular overlap 100 $\pm$ 0% of the time (purple) and the inferior position [azi = 0, ele = -10] (cyan) fell in the binocular zone 97 $\pm$ 3% of the time.

3) Is it possible that the decreased scatter within the binocular zone is simply due to binocular matching together with a larger cortical magnification? What would happen, for example, if the authors were to repeat the experiments on mice reared with mis-aligned eyes (presumably yielding only monocular neurons)? Will the scatter difference disappear?

This is an interesting suggestion. If reduced RF scatter was only the result of binocularity then both RF scatter and pRF sizes would form a step-function, being lower in binocular regions of cortex and higher in monocular regions. We have observed that both pRF size and single-cell scatter form continuous functions (for example, the 3D plot in Figure 1g shows a continuous relationship with both azimuth and elevation, without a change in the slope at the boundary between binocular and monocular regions of the visual field). More formally, we have examined the explained variance of regression models using a continuous predictor based on r-eccentricity vs. models based only on binocularity (Figure R3). The continuous models outperform the discrete binocular models, both in explaining pRF size in wide-field data and in explaining RF scatter in the two-photon data. For the wide-field data the continuous model had a higher r^2 value for all 22 hemispheres tested and explained 81% of the variance of pRF size, whereas the binocular-model only explained 37% (paired t-test, $p \ll 0.001$). For the two-photon data we examined whether the scatter of the residuals of the cortical magnification function were greater for monocular compared to binocular regions but we found no such relationship (bootstrap test, all three mice $p > 0.05$). These analyses have been added to the manuscript on lines 79-83, lines 137-139 and Supplementary Fig. 3b.

We cannot rule out that there was a contribution of binocularity to the reduced scatter at the focea. However, the analyses above indicates that the contribution of binocularity must be small. Indeed, the increase of pRF size with r-eccentricy also occurs in the monocular regions. It is therefore very likely that the same effect is present in strabismic mice. We have extended the section in the discussion related to binocularity and discussed these issues in the revised manuscript on lines 282-286.

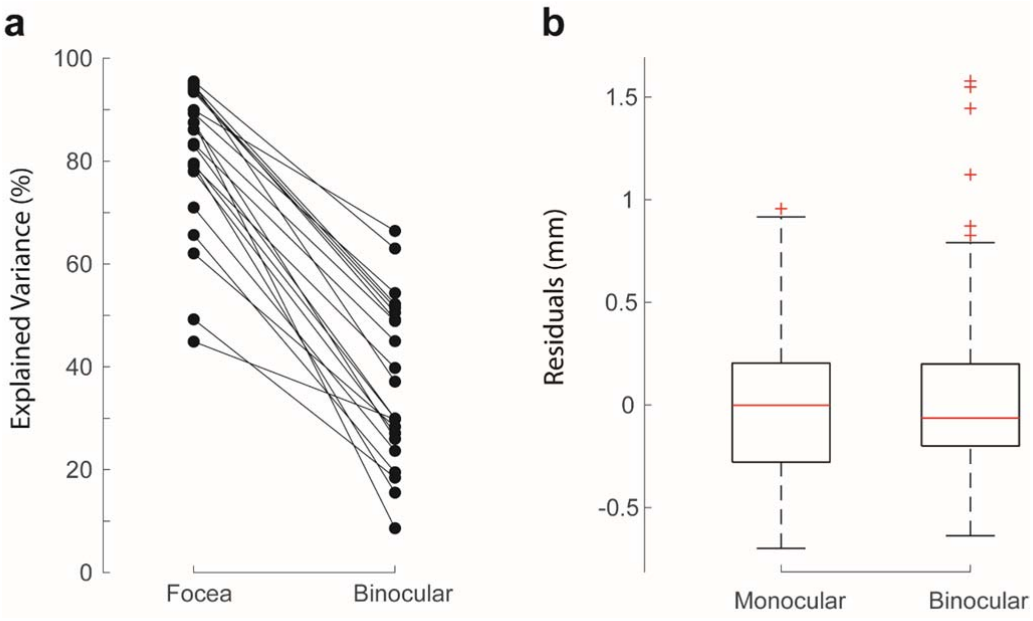

Figure R3. a r-Eccentricity better explains pRF sizes than binocularity. **a** Explained variance of a regression model with a predictor containing the angular distance of each pRF from the focea (labelled 'focea') and a model in which the predictor was categorical, i.e. RF size was a set function with one value for monocular pRF positions and another value for binocular pRF positions. Each dot is a hemisphere (n = 22). The r-eccentricity model explained more variance than the binocular model in every hemisphere. **b** The pattern of residuals around cortical magnification function fits could not be explained by binocularity. The boxplots show the distribution of residuals from cortical magnification function fits (e.g. Figure 4b). If RF scatter was reduced in binocular regions, the spread of the residuals distribution would be lower than in monocular regions. The boxplots show data from mouse M1, it can be seen that the distributions were comparable. A bootstrap statistic, comparing the standard deviation of the two distributions against a scrambled dataset, showed no significant differences between the two distributions for all three animals.

Altogether, the non-uniformity of scatter of RFs in V1, along with its consequences for the organization of downstream areas and behavior are an important contribution to the field.

Reviewer #2 (Remarks to the Author):

This manuscript elegantly synthesizes multiple methods, including widefield imaging, electrophysiology, 2-photon calcium imaging, and computational modeling, to provide a wholistic description of the relationship between mouse retinotopic organization, receptive field size and scatter across single cell and population measures. Importantly, this combination of techniques demonstrates a novel finding of a region of increased spatial resolution in the visual field directly in front of and above the mouse, the focea. The authors find a reduction in population receptive field (pRF) size at the focea, due to an increasingly ordered representation of visual space with reduced scatter in single cell RF positions in this region, beyond an increase in cortical magnification, rather than a reduction in single cell RF size as one might naively expect.

The results described in Figures 1-5 demonstrate the existence of the focea with widefield imaging, then go on to show with electrophysiology and single cell 2-photon calcium imaging that the reduction in population receptive field size at the focea is not due to a reduction in single cell receptive field size, but rather is associated with reduced scatter in RF position as well as an increase in the amount of cortical territory dedicated to this part of space. The results are quite convincing, especially given the large size of the widefield and electrophysiological datasets. In addition, these results provide important insight into the nature of the widefield signal and pRF measures generally, demonstrating that population RF sizes measured with this method arise from scatter in both cellular and neuropil signals over a 200-400um region, whereas signals from cell bodies (from single cell 2-photon or MUA with ephys) only cannot fully account for the observed pRF sizes in widefield data. This is a valuable insight for the field and should be considered in the interpretation of widefield results and in any assumptions of how widefield signals translate to single cell properties.

In contrast to the lack of systematic changes in single cell RF size across V1, in figure 6 the authors demonstrate a relationship between single receptive field size and eccentricity from the focea in several higher visual areas. The authors suggest that the reduction in population RF size in V1 and associated reduction in RF scatter could translate to smaller RFs in higher visual areas, contributing to higher spatial resolution vision in the focea. The mechanisms of such a transformation remain unexplored.

The authors move beyond characterization and delve into the functional implications of the measured relationships during freely moving and trained behaviors. Using a visual detection task, the authors demonstrate increased spatial acuity in the foceal region of space, marked by higher spatial frequency thresholds (and thus increased resolution) for stimuli directly in front of and slightly above the mouse. These results confirm the behavioral relevance of their physiological results and are a key component of the paper. Using a newly developed system for tracking head and eye movements in freely moving mice, they show that mice use compensatory eye movements to stabilize the focea on the region of space directly in front of and slightly above the mouse. This was true in multiple diverse behavioral contexts including exploration of an open field, social interaction, and object tracking on a screen. The authors suggest that the utility of keeping the focea stable in this location is to facilitate accurate object identification and navigation in the context of optic flow during locomotion.

Overall, the experiments are rigorously performed, well grounded in the literature, include

appropriate controls, and are clearly described with compelling visualizations and analysis. The combination of functional and behavioral measures, along with modeling and thorough computational analysis, provides a comprehensive view of the structure and function of a focea for high acuity vision in the mouse, including evidence for the behavioral utility of the focea. These results clearly provide a valuable addition to our understanding of mouse visual cortex. I recommend that the manuscript be accepted with minor revisions, per the specific comments below.

We would like to thank the reviewer for their careful reading of the manuscript and their positive comments.

Specific comments

General

The authors may wish to reference these relevant studies and discuss how their results relate to previous findings:

Michaiel, A. M., Abe, E. T. T. & Niell, C. M. Dynamics of gaze control during prey capture in freely moving mice. Elife 9, 1–27 (2020). https://elifesciences.org/articles/57458

We have added this reference and summarized the main finding on lines 332-334.

Oommen, B. S. & Stahl, J. S. Eye orientation during static tilts and its relationship to spontaneous head pitch in the laboratory mouse. Brain Res. 1193, 57–66 (2008). https://pubmed.ncbi.nlm.nih.gov/18178173/

We have added a reference to this study on line 327.

Waters, J. et al. Biological variation in the sizes, shapes and locations of visual cortical areas in the mouse. PLoS One 14, 1–13 (2019). https://journals.plos.org/plosone/article?id=10.1371/journal.pone.0213924

We have referenced this paper on the section of map realignment on lines 823-826.

Zhuang, J. et al. An extended retinotopic map of mouse cortex. Elife 6, (2017). https://elifesciences.org/articles/18372

Regarding this last publication, one interesting point of discussion is the implication of the reduced scatter and pRF size at the focea and the organization of areal boundaries in this zone. As demonstrated in Figure 1 as well as in Zhuang et al. (Figures 4&5), the boundary between V1, LM, AL, and RL does not always come to a clear meeting point, and the lateral V1 boundary measured with functional imaging does not match the anatomical boundary of V1 measured with cytoarchitecture or histological markers. How might the size and scatter of RF locations in the binocular zone of V1 and neighboring areas relate to the ability to accurately map areal boundaries with widefield imaging, and to anatomical boundaries based on cellular architecture and connectivity?

We have now referenced this paper on line 310. It is an interesting observation that the lateral boundary of V1, as defined by histological markers, extends beyond the boundary defined by the reversal in retinotopy. We note that Zhuang et al show that the location of the V1/LM boundary defined by wide-field imaging agrees very well with the boundary defined by the reversal in azimuth at the single-cell level using two-photon imaging. The boundaries that we defined using wide-field imaging are therefore an accurate representation of the extent of the retinotopic V1 map and we observed the smallest pRFs close to the lateral border of V1. Zhuang et al. discuss several explanations for the discrepancy between retinotopy and the architectonic boundary. In this context it is of interest that the regions with unexpectedly increased cytochrome-oxidase activity may coincide with the weak dLGN projections to LM (Antonini et al., 1999; Oh et al., 2014). Here we focused on pRF-size and retinotopy and we did not measure cytoarchitectonic markers and prefer to refrain from an extensive discussion of the discrepancy between the cytoarchitectonic and retinotopic boundaries.

Main text and figures

• The text is very well written and clear, particularly in the description of results for Figures 1-5. Results and discussion for Figures 6-8 regarding functional implications of the foceal representation could be elaborated to provide additional context and motivation for these experiments, how they relate to the findings in Figures 1-5, and their practical significance. The inclusion of behavior is very valuable to the study and it's impact should be emphasized.

We have elaborated on the sections describing the last three figures. Please see our replies to comments below. In particular, we have extended the discussion of the results from the higher visual areas and the behaviour on lines 301-319.

• Throughout the text, ensure that references to population RFs vs. single cell RFs are abundantly clear, as they can easily be confused and the interpretation is very different depending on which is being discussed.

We improved the descriptions of pRFs and single cell RFs as suggested by the reviewer.

• Keep in mind that the various spatial reference frames and transformations between them can be difficult to keep track of for those not familiar with these types of experiments, especially for eye tracking in freely moving animals. Clarify any relevant text as necessary.

We have endeavoured to write as clearly as possible about the different reference frames and have clarified the text on lines 208-227 to make it clear how the focea was defined in eye coordinates and how this was related to azimuth/elevation co-ordinates used in the head-fixed experiments.

• Line 64, and generally – why did the authors choose to distinguish the focea from the fovea of primates? Why use the term 'focea'? Some context would be helpful to the reader here. Are there other species with a focea? Is this a novel term or is there precedence for its use?

The term 'focea' is a new term which we have coined to distinguish the organisation of mouse visual cortex from that of primates. The fovea strictly refers to a region of higher photoreceptor density in the primate retina, similarly 'area centralis' refers to a region of high photoreceptor density in some

other species like the cat (Rapaport and Stone, 1984). In mice this organisation of the retina is largely absent and we do not want to imply any retinal specialisation in this study. Partly we chose the term 'focea' (from the latin 'to focus') for convenience as continually referring to 'a point at 0° azimuth and 20° elevation' becomes cumbersome, but also because we wish to convey that mice have a cortical specialisation which enhances processing of this region of the visual scene. We have now introduced the term focea more carefully, on line 64-67.

• Line 67 – please elaborate on description of r-Eccentricity and how it is calculated based on altitude and elevation.

We have now added a description of the calculation of r-Eccentricity to the manuscript on lines 69-71: "…r-eccentricity is the angle between the center of the pRF and the 0° azimuth, 20° elevation point, in a spherical co-ordinate system centered on the mouse, see Methods".

We have also revised the equations on line 800-801 for clarity.

• Figure 1 – it would be valuable to provide the retinotopic maps for all mice recorded as a supplemental figure, so that the reader can gain an intuition for the variability in the size, shape, and layout of the areas (ex: Waters et al., 2019).

We have now included a new supplementary figure (Supplementary Fig. 2) showing retinotopic maps (field-sign maps) and pRF sizes for 15 unilaterally imaged mice. It is included here as Figure R4 for the reviewer's convenience (next page).

• Figure 4d – please describe r-Eccentricity in the figure legend as this is the first time it is mentioned in this figure.

We have added a description of r-Eccentricity to Figure 4d, and also Figures 1h and 2c.

• Line 111 – states that mice that were imaged were injected with AAV-GCaMP6f, however the first section of the methods states that Thy1-GCaMP6f mice were used for 2-photon experiments

Thanks for pointing out this mistake – the experiments in which we tiled V1 were run with non-injected Thy1-GCaMP6f mice, whereas for the experiments in which we imaged cells in higher areas we first identified the location of the different visual areas using pRF mapping and then targeted these regions with AAV-GCaMP6f injections to amplify the GCaMP signal.

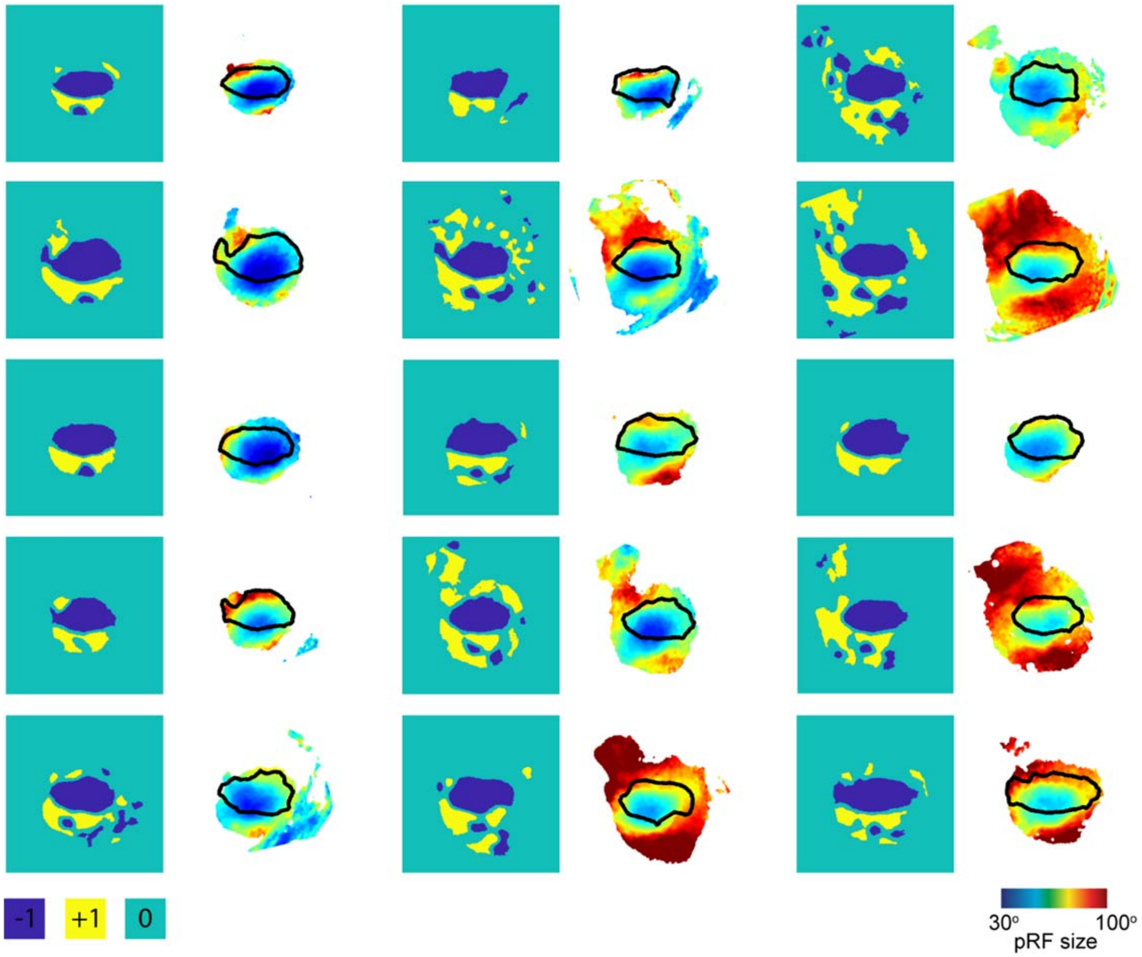

Figure R4. *Retinotopic maps from 15 unilaterally imaged mice. For each mouse the field-sign map derived from the wide-field pRF maps is shown on the left and the map of pRF size on the right. Area V1 is marked on the pRF size maps with a black outline. All maps were thresholded by the quality of the pRF model fit (at Pearson's r = 0.75).*

• Line 136 – typo, I believe that "included" shoud be "including" here

Thanks for spotting this typo.

• Figure 5a, I believe the title for the far right panel should read "RF size" rather than "pRF size" as the measure is per pixel of the image rather than an aggregate over a window.

We agree with the reviewer and to avoid confusion with the later analyses of populations of pixels we have changed the title to be 'RF Size'.

• Figures 4 and 5 – it would be good to show a quantification of RF size as a function of azimuth, elevation and r-Eccentricity for the 2-photon data, similar to what is shown in figure 2C for the electrophysiology data, to confirm that RF sizes are not changing drastically with distance from the focea. For instance in Figure 5a, right panel, there does appear to be some variation in RF size in portions of the image. It would be helpful to know whether there is any systematic relationship to

retinotopic position, and whether any effect is consistent across mice. This could be a supplemental figure.

We have added a supplementary figure (Supplementary Fig. 5) showing the individual cell/pixel data in the same format as Fig. 2c for the two-photon data. There were no clear relationships between pRF/RF size and azimuth/elevation/r-eccentricity. The figure is reproduced here as Figure R5 for the reviewer's convenience.

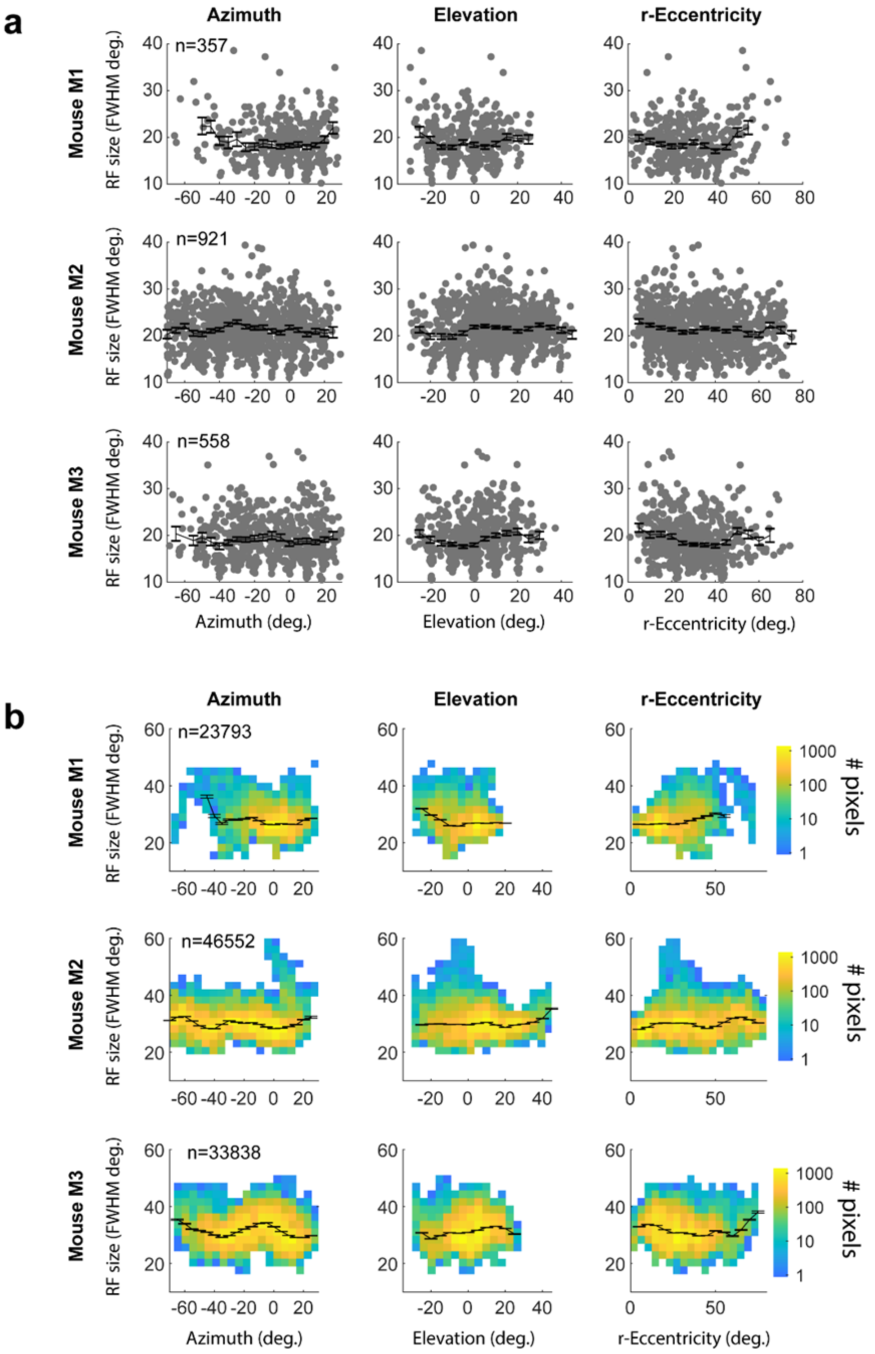

Figure R5. a *There was no consistent relationship between the azimuth (left panel), elevation (middle panel) and r-eccentricity (right panel) of the RF of individual cells and the RF size in the V1 two-photon imaging data in the three imaged mice. Data shown with the same format as Figure 2c.* ***b*** *No*

consistent relationships were found for RF size of pixels of the raw images and the azimuth, elevation or eccentricity of the raw image RF. Due to the high number of data-points they are shown here as density plots on a logarithmic scale.

• Figure 5 – intermediate plots for the scatter analysis, such as the residuals as a function of eccentricity, akin to what is shown in Figure 4d, could be provided as supplemental material to support the summarized results for the pixel-wise analysis in Figure 5b&c.

Our aim in Figure 5 was to investigate the difference in the slope of the relationship between r-eccentricity and pRF size between the wide-field data and the single-cell data. The analyses in Fig. 5b,c are therefore not comparable to the analysis of residuals shown in Fig. 4d (which tests the influence of cortical magnification) but to the analysis of pRF size and the role of scatter in RF positions (see Fig. 4g,h). The intermediate steps in the analysis of Figure 5 are the computation of the slopes, and this computation has been illustrated with insets in Fig. 5c.

• Line 160-161 – please elaborate on why one might predict that decreased scatter in the focea would result in smaller RFs in downstream areas and why this is of functional significance, to provide more context to the reader.

We have now added a section explaining the logic of why decreased scatter in V1 could lead to smaller RFs in higher visual areas on lines 176-179 and we have added an explanatory panel to Figure 6 (Fig. 6a).

• Line 170 – specify whether “reduced RF size” refers to single cell RF size or population RF size, as these have different implications for the question at hand.

We have specified that we are referring to single-cell RFs at this point.

• The implications of the results shown in Figure 6 should be more thoroughly described in the results and in the discussion. The idea that reduced scatter in V1 is converted to smaller RFs and higher spatial resolution in higher visual areas is intriguing, but not conclusively demonstrated by the results presented. As such, the conditions that would need to be present to give rise to this situation (i.e. the sampling strategy of neurons in higher visual areas) should be discussed to provide context to the reader, and to provide suggestions for future experiments to confirm these predictions.

Thanks for pointing this out. We have now added a section explaining the logic of why decreased scatter in V1 could lead to smaller RFs in higher visual areas on lines 176-179 and in Fig. 6a. We have also discussed the ramifications of these results in a section from line 301-313.

• In addition, the result that LM, AL and AM show this relationship between RF size and eccentricity, but RL and PM do not, should be further discussed. Why might some areas require better acuity at the focea and others do not? What does this suggest for the functional role of those areas?

We have added discussion of this point to lines 308-313. We also note a mistake in the original manuscript, the bootstrapped reliability index that we use to exclude unreliable RFs was mistakenly not applied to these data. We have corrected this error in the revised manuscript in Figure 6c. The results remain the same, with the exception of area AM, which no longer shows a significant

relationship between r-Eccentricity and RF size. The lack of a relationship in areas RL, AM and PM may stem from the retinotopy of these areas. Areas RL and AM are biased towards representations of the inferior visual field and PM is biased towards temporal regions. These regions therefore do not contain an extensive representation of the focea unlike LM and AL, which do sample the foceal region of V1.

• Figure 7b – it would be good to show the full spatial frequency detection curves for all mice in the study, in addition to or rather than a single example. If these results are too noisy, adding additional mice is suggested.

The full spatial frequency detection curves are now shown in Supplementary Fig. 7. They are reproduced here in Figure R6 for convenience.

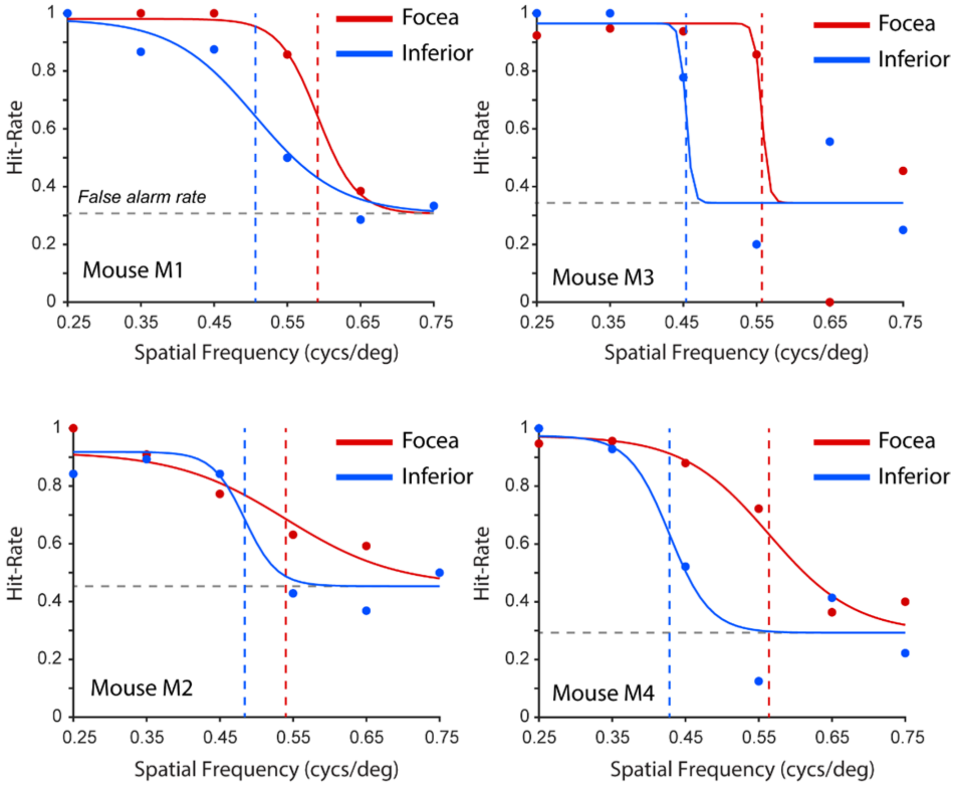

Figure R6. *Spatial frequency detection curves for all four mice. The data-points are the average hit-rates across sessions for each tested spatial frequency. Acuity at the focea was higher (spatial frequency threshold is indicated by the red dashed line) than at the position in the inferior visual field (dashed blue line). The curves are fits of logistic functions as described in the main text.*

• Results are only shown for one mouse in Figure 8. Results from all mice should be reported, especially for the results in Figure 8b.

We have now included the distributions of the focea position for the other mice in Supplementary Fig. 8b.

• Please add a legend for bar colors to Supplemental Figure 4B for clarity.

These have been added – the figure panel became part of Figure 8 of the main text (see next point).

• One of the more unique and valuable aspects of this work is the measurement of eye position across multiple behavioral contexts as it relates to the focea, however these results are buried in the supplemental matrial and are not as thoroughly explored as they could be. Supplemental Figure 4B should be moved to the main figures (Figure 8). Additional analysis of eye position in these various context would also be valuable. For example, how do head and eye position change as the mouse approaches another mouse? The dynamics of these interactions are interesting and the data is there.

We followed the advice and moved the eye position data for different behavioral contexts to Figure 8.

We investigated the coupling between head and eye movements during the different behavioral contexts in detail in a previous study (Meyer et al., 2020). Briefly, eye movements in mice are tightly coupled to the head but in two different ways (related to head orientation and to head yaw rotation). Both types of eye-head coupling are preserved during the different behavioral contexts, including the behaviors investigated in the present manuscript (free exploration of an open field environment, social interaction, object tracking). Consequently, changes in eye position during social interaction are largely determined by an animal's head orientation and rotation

We agree that a detailed description of approach dynamics during social interaction would be very valuable in itself. However, such an analysis would require a precise description of body and head dynamics of both mice during interaction bouts, including identification and classification of those bouts. While recent progress in computer-based analysis of behavior from video facilitates extraction of such variables (e.g., Segalin et al., bioRxiv (2020)), our data come with the additional challenge of camera cables and IR illumination of the head required for eye tracking. The development and refinement of methods that can deal with such scenarios is beyond the scope of the current manuscript.

• Images depicting the experimental conditions for open field and social interaction should be provided in addition to the representation of object tracking in Supplemental Figure 4C, to provide context for Supplemental Figure 4B. Again, all relevant figure panels to this analysis would be better positioned in the main figures.

We have now included example images from an overhead camera for the open field and social interaction in Supplemental Fig. 8c.

Methods:

• Overall, very thorough description of methods used, all very rigorous, well controlled, and consistent with previous literature.

Thanks for this positive assessment.

• Throughout the methods, it would be helpful for novice readers if the equations were described in words, in addition to the formula being provided.

We have added explanatory text to several of the equations in the methods.

• Line 647 – which painkillers were provided?

Metacam (5mg/kg).

• Line 651 – I believe the authors meant to say “as described below” rather than “above” in reference to methods for pRF mapping

Thanks for spotting this typo.

• Lines 651-654, the authors describe injecting animals with AAV prior to imaging higher visual areas with 2-photon calcium imaging, however in the “Animals” section of the Methods, they describe using Thy1-GCaMP6f mice for the 2-photon imaging experiments. Please clarify which methods were used.

We have now clarified this issue. For all two-photon experiments we used Thy1-GCamP6f mice. For the experiments in which we tiled V1 we imaged the genetically expressed calcium signal. For experiments in which we imaged higher areas we also injected AAV-GCaMP6f into identified higher areas to amplify the calcium signal.

• Line 665 – “Hz” is repeated twice. Also the authors state that pupil was tracked at “50/100Hz”, do they mean either 50 or 100Hz?

We have corrected the typo. And the reviewer is correct, early sessions were recorded at 100Hz, but the recording system struggled to store data at this rate and so in later sessions the eye-data were recorded at 50Hz.

• If the authors tracked the pupil during wide field imaging with the head level, did they observe that the center of gaze was 20 degrees above the horizon (as in Oommen & Stahl, 2008)? Can they report their eye position measurements for these experiments?

The eye-tracking system used in our wide-field set-up was primarily used to identify periods of eye-movement so that these could be removed or regressed out of the pRF mapping analysis. We could not relate the eye position to a real-world gaze angle with this system.

• Line 667 is a bit confusing– “to ensure that check-size in degrees of visual angle was constant”, perhaps it could be rephrased as “to ensure that the size of the check in degrees…”

We have adopted the reviewer’s suggestion here.

• Why not use an open source spike detection algorithm, such as Kilosort, instead of thresholding?

In our hands the yield obtained from spike-sorting signals obtained with NeuroNexus A16 probes is rather low and largely consists of multi-units with only occasional well-isolated units. The degree of pooling across cells using electrophysiology was small enough to cause the size V1 spiking RFs to hardly vary with eccentricity. This result is in accordance with the two-photon data in V1.

• Line 943 – specify DeepLabCut in the text in addition to citing the reference

This has been added.

• Line 907 - how is the focea defined? This is described in the results but would be helpful to summarize here.

We have added the definition.

• I do wonder why the authors did not do an analysis of eye torsion with their pupil tracking data in the freely moving animals, similar to what was done in ref. 52. Perhaps there was some barrier to doing this analysis properly? If not, it could be suggested that they include that analysis and use directly measured torsion values. It seems they should have the necessary data to do this analysis.

Eye torsion measurements in mice (in particular freely moving mice) are very challenging for two reasons. First, the only feature for optical tracking of eye torsion is the slightly uneven pupil margin. While it is possible to track a small number of small-scale features on the margin, these features are currently hard to identify when the pupil is small (e.g., due to bright light), occluded by the eyelid when mice are making eye movements or when the pupil is large. Second, the number of features that can be tracked reliably is largely limited by the camera lens. Similar to work in rats (Wallace et al., 2013), tracking more features would enable reliable characterization of eye torsion in freely moving mice. However, due to the comparably small size of the mouse (~25 grams vs ~250 grams for rats) the lens must be very lightweight. The current lens is ~0.1 grams. We have not yet found a lens that is lightweight enough for our purposes but still provides high-enough resolution to reliably track multiple small-scale features. However, eye torsion measurement in freely moving mice is certainly something we hope to achieve in future work. We have added a sentence to the methods to clarify the reasons for performing the torsion measurement in head-fixed animals. We also note that the only available torsion measurement in freely moving rats (torsion gain ~0.3; Figure 2F in Wallace et al. (2013)) gave results that were similar to our measurements in head-fixed mice.

• In addition, I can think of a few ways that the authors could provide increased confidence in their torsion measurements and the overall approach. First, they could perform the measurement of torsion in multiple head fixed mice, rather than just one, to give a sense of the variability across mice in Supplemental Figure 4a and confirm that the relationship is always linear and that the slope is similar across mice. Second, they could re-evaluate their model with varying values of the slope of the relationship of eye torsion to head pitch, such as 0.2 or 0.4, in addition to their used value of 0.3, to assess how sensitive their results are to this value. If slight variation in the slope used in the model does not significantly impact their results and interpretation, it would give confidence that their approach is not a major confound.

We thank the reviewer for these suggestions. We repeated the same experiment in a second mouse and found similar results (torsion gain 0.35 compared to 0.30 for the first mice) and included the data in Supplemental Fig. 8a. All analyses in the revised manuscript were updated to use the average torsion gain value (0.325).

We also repeated the analysis with a torsion gain of 0.217 (-33%) and 0.433 (+33%), respectively (Supplemental Fig. 8d in revised manuscript). The precise torsion gain value had a small influence on

focea elevation in freely moving mice: a change in torsion gain of 33% resulted in a 10% change in focea elevation.

• I noticed that they may have used the wrong reference on line 959 of the methods – here they refer to ref. 53 regarding torsion measurements in rats, but I believe those measurements were actually done in ref 52.

We thank the reviewer for spotting this: we have now corrected the reference.

References:

Antonini, A., Fagiolini, M., Stryker, M.P., 1999. Anatomical correlates of functional plasticity in mouse visual cortex. J. Neurosci. 19, 4388–4406.

Meyer, A.F., O'Keefe, J., Poort, J., 2020. Two Distinct Types of Eye-Head Coupling in Freely Moving Mice. Curr. Biol. 2020.02.20.957712.

Oh, S.W., Harris, J.A., Ng, L., Winslow, B., Cain, N., Mihalas, S., Wang, Q., Lau, C., Kuan, L., Henry, A.M., Mortrud, M.T., Ouellette, B., Nguyen, T.N., Sorensen, S.A., Slaughterbeck, C.R., Wakeman, W., Li, Y., Feng, D., Ho, A., Nicholas, E., Hirokawa, K.E., Bohn, P., Joines, K.M., Peng, H., Hawrylycz, M.J., Phillips, J.W., Hohmann, J.G., Wohnoutka, P., Gerfen, C.R., Koch, C., Bernard, A., Dang, C., Jones, A.R., Zeng, H., 2014. A mesoscale connectome of the mouse brain. Nature 508, 207–214.

Rapaport, D.H., Stone, J., 1984. The area centralis of the retina in the cat and other mammals: focal point for function and development of the visual system. Neuroscience 11, 289–301.

Wallace, D.J., Greenberg, D.S., Sawinski, J., Rulla, S., Notaro, G., Kerr, J.N.D., 2013. Rats maintain an overhead binocular field at the expense of constant fusion. Nature 498, 65–69.

Reviewer #1 (Remarks to the Author):

The authors have responded to my concerns and questions satisfactorily. I have no further comments.

Reviewer #2 (Remarks to the Author):

The authors did an excellent job of addressing all concerns. I am happy to recommend this manuscript for publication. This work is a valuable addition to the field, providing novel insights with important implications about the function of higher visual areas in the mouse. I look forward to seeing how it is received by the neuroscience community.

One very minor suggestion - In Supplementary Figure 2, rotate visual maps so that the anterior direction is pointing upwards, and/or add arrows to indicate anatomical direction (as in Figure 1E).

REVIEWERS' COMMENTS

Reviewer #1 (Remarks to the Author):

The authors have responded to my concerns and questions satisfactorily. I have no further comments.

We thank the reviewer for their positive response.

Reviewer #2 (Remarks to the Author):

The authors did an excellent job of addressing all concerns. I am happy to recommend this manuscript for publication. This work is a valuable addition to the field, providing novel insights with important implications about the function of higher visual areas in the mouse. I look forward to seeing how it is received by the neuroscience community.

We thank the reviewer for their positive response.

One very minor suggestion - In Supplementary Figure 2, rotate visual maps so that the anterior direction is pointing upwards, and/or add arrows to indicate anatomical direction (as in Figure 1E).

We have implemented the suggested changes in Supplementary Figure 2.
